# Supplementary material for: Machine learning-based extrachromosomal DNA identification in large-scale cohorts reveals its clinical implications in cancer
Source: Nat Commun. 2024 Feb 19;15:1515. doi: 10.1038/s41467-024-45479-6 (PMC10876971; doi:10.1038/s41467-024-45479-6)
Supplement: Supplementary file 1 — Supplementary Information [file 41467_2024_45479_MOESM1_ESM.pdf]

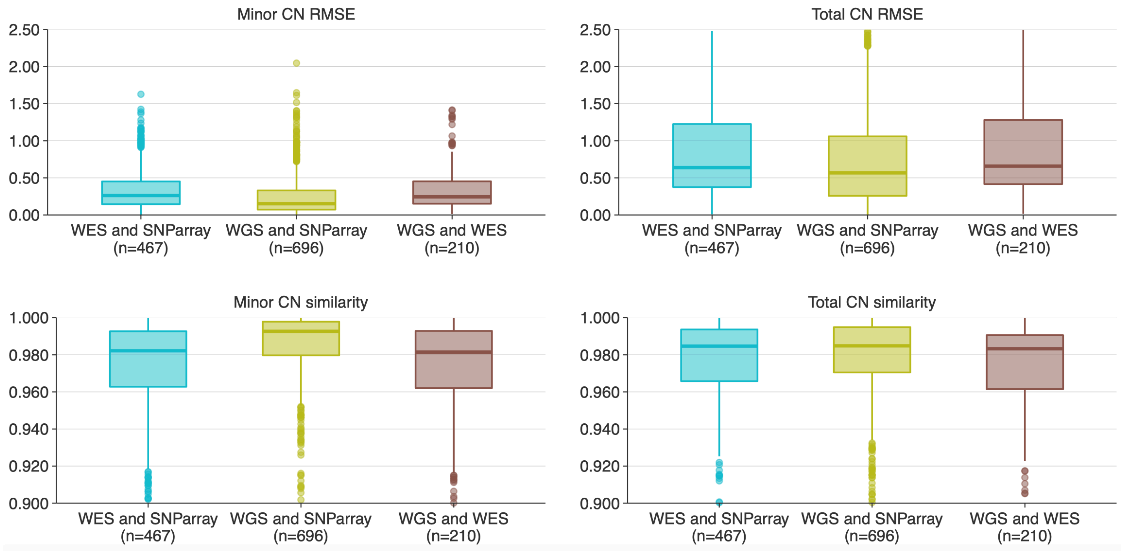

Supplementary Figure 1 | Allele-specific copy number calling with data from different high-throughput genomics is comparable.

Allele-specific copy number profiles (including total copy number and minor copy number) comparison between different high-throughput genomic assays (WES, WGS and SNP array) with RMSE (root mean square error, lower is better) and cosine similarity (higher is better) approaches, respectively. Abbr.: CN, copy number.

Supplementary Figure 2

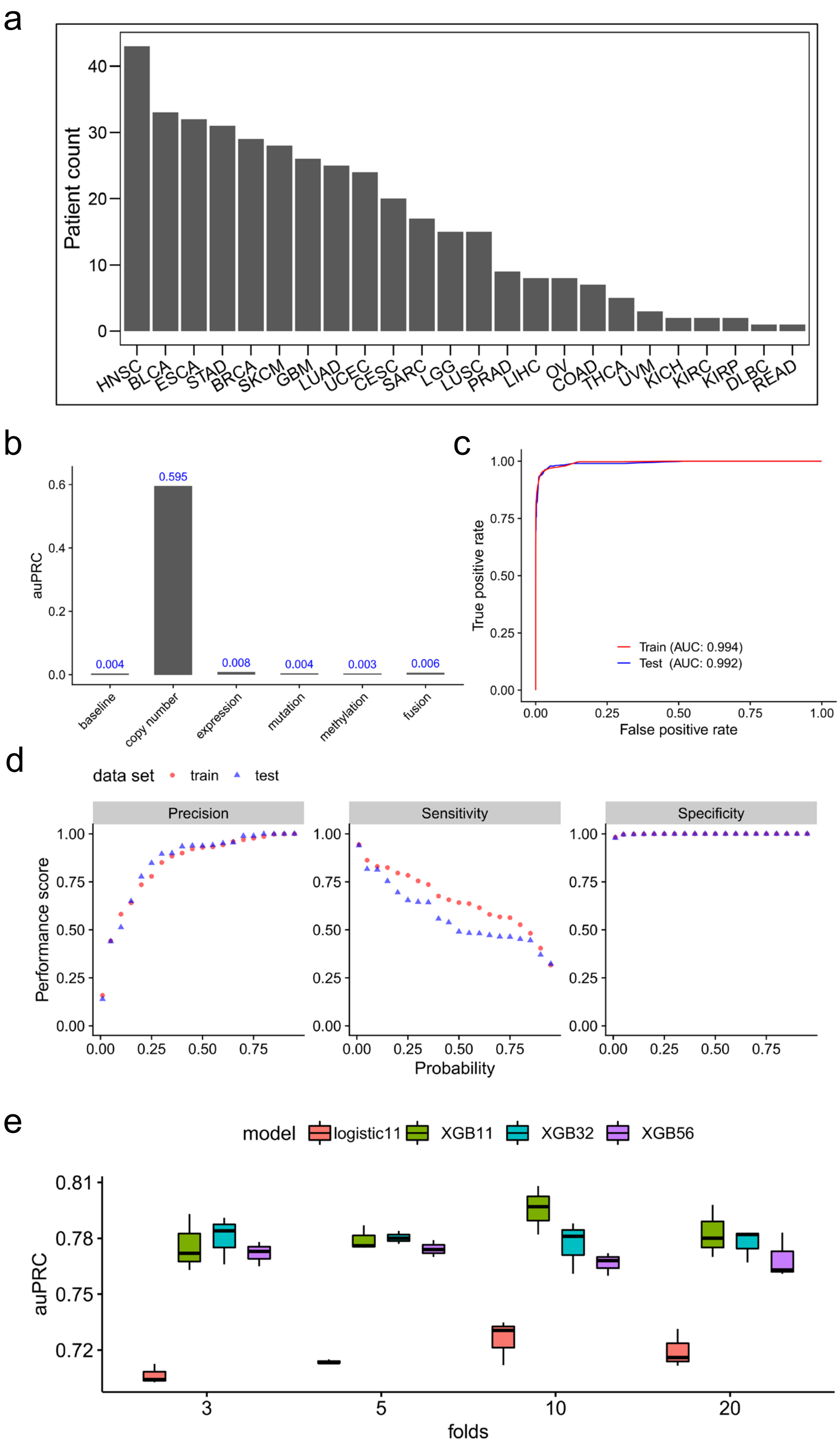

Supplementary Figure 2 | ecDNA cargo gene prediction modeling and performance estimation.

a, Cancer type distribution of 386 TCGA cancer patients for modeling. TCGA cancer type abbreviations are explained at https://gdc.cancer.gov/resources-tcga-users/tcga-code-tables/tcga-study-abbreviations. **b**, Area under precision-recall curve values of exploratory ecDNA cargo gene prediction logistic regression models by different gene-level molecular profiles. **c**, Receiver operating characteristic curves and corresponding AUC values on training set (90%) and testing set (10%). **d**, Precision, sensitivity, and specificity trends on training set (90%) and testing set (10%) under different probability threshold for classification. **e**, Performance comparison between ecDNA cargo gene prediction models generated from different strategies (logistic regression and XGBOOST) and different feature numbers (11, 32 and 56) by different k-fold (k is 3, 5, 10, 20, respectively) cross-validations in 3 repeats. 11 features represent a basic feature set, 32 features represent the 11 features plus 19 copy number signatures, and 56 features represent the 32 features plus 24 cancer types the modeling tumor samples belong to.

CRC1002 – MYC/Chr8 centromere/DAPI

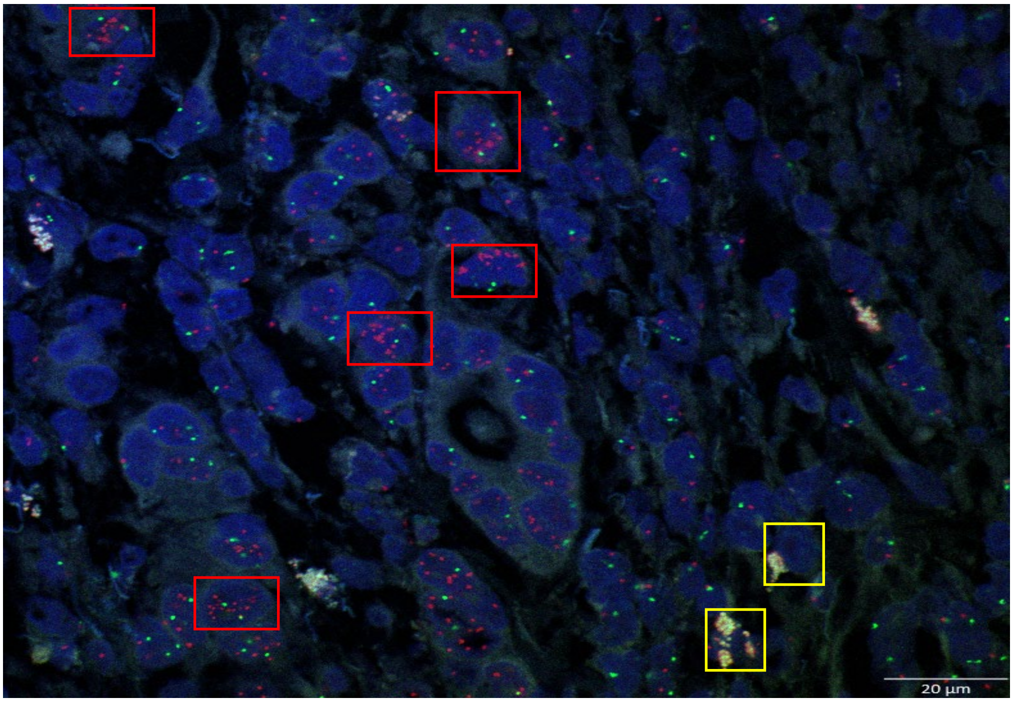

CRC1057 – ERBB2/Chr17 centromere/DAPI

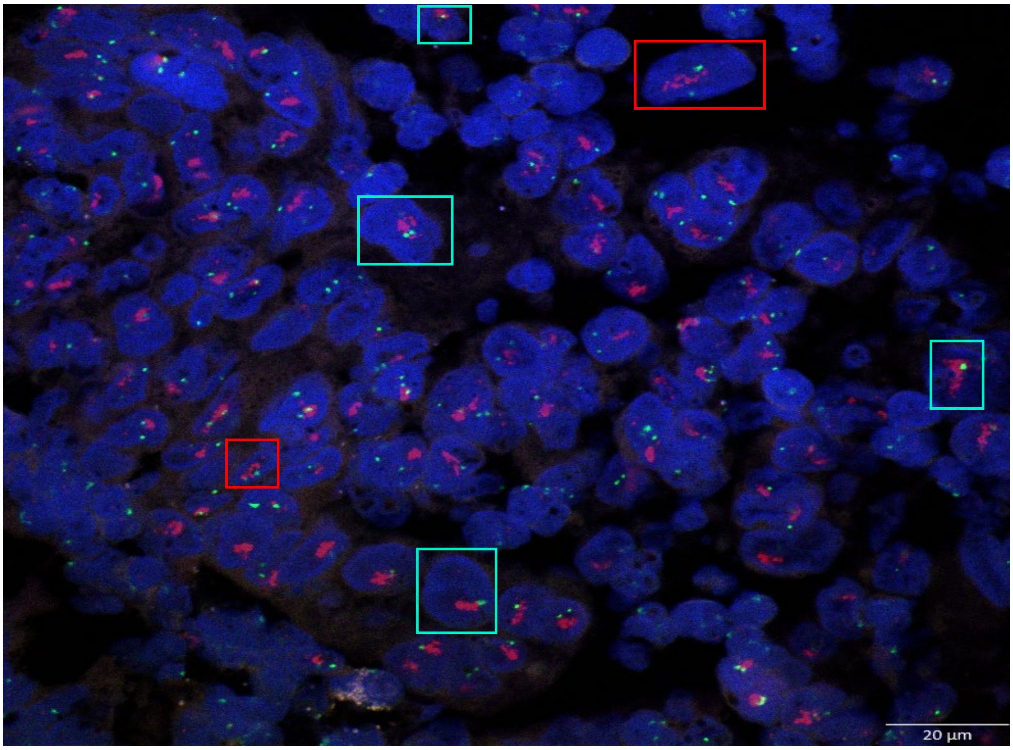

Supplementary Figure 3 | FFPE DNA FISH representative images of two CRC tumor samples from the Changkang Project.

Some regions are highlighted by different colored boxes. Representative regions with diffuse gene amplification signal are highlighted by red box in the patient CRC1002. Regions with similar pattern observed in the patient CRC1057 are also labeled with red box. Regions with co-amplification pattern of gene *MYC* and chromosome 8 centromere observed in the patient CRC1002 are highlighted by yellow box. Representative regions with gene clustered amplification pattern observed in the patient CRC1057 are highlighted by cyan box.

Supplementary Figure 4

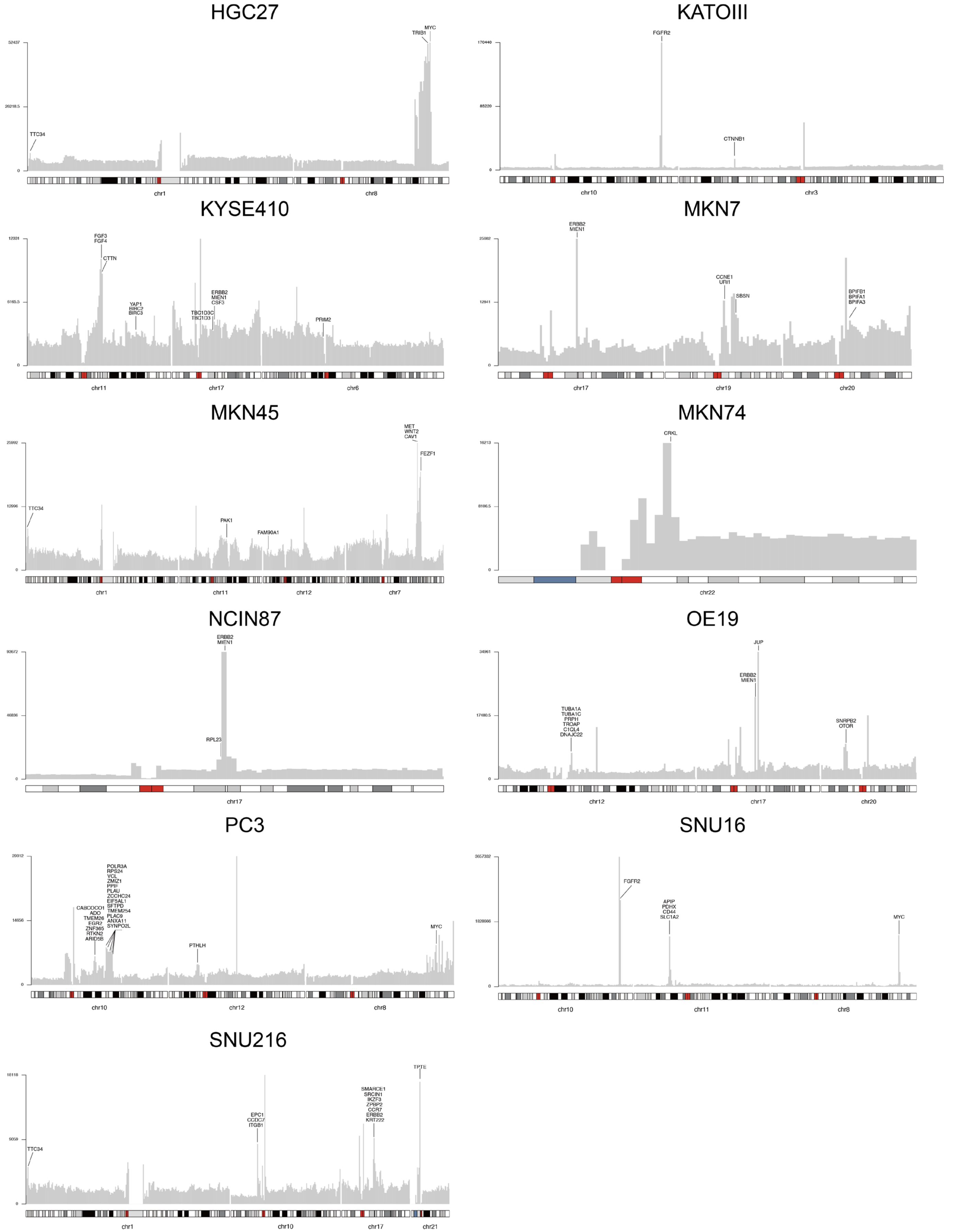

Supplementary Figure 4 | Validating GCAP predictions using Circle-Seq in cancer cell lines.

Labeled ecDNA cargo genes predicted by GCAP are depicted. The y-axis represents Circle-Seq read density, measured as the number of reads overlapping every one-megabase window.

circular+ (n = 164) v/s circular− (n = 831)

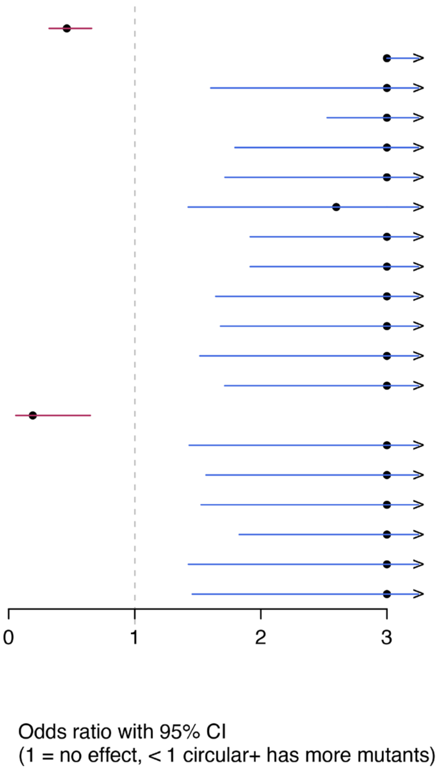

	circular+	circular−	OR	P−value
TP53	101	353	0.461	***
MUC12	0	58	Inf	***
PIK3CA	8	122	3.353	***
SPTBN4	0	47	Inf	***
AHNAK2	3	78	5.553	***
KMT2D	3	75	5.319	***
MUC16	13	152	2.598	***
BMPR2	1	54	11.315	**
DYSF	1	54	11.315	**
PRKDC	1	47	9.761	**
ANKRD11	1	48	9.981	**
DNAH2	2	56	5.847	**
PRR12	0	33	Inf	**
IGHV1OR21−1	7	7	0.191	**
FSIP2	4	74	3.907	**
COL14A1	1	45	9.322	**
NOTCH3	1	44	9.103	**
KDM6B	0	35	Inf	**
TACC2	2	53	5.513	**
PKD1	2	54	5.624	**

Supplementary Figure 5 | The gene list displays mutations that differ between circular+ and circular- patient groups in the SYSUCC CRC cohort. Only genes with *P*<0.005 are shown. **P*<0.05, ***P*<0.01, ****P*<0.001.

Supplementary Figure 6

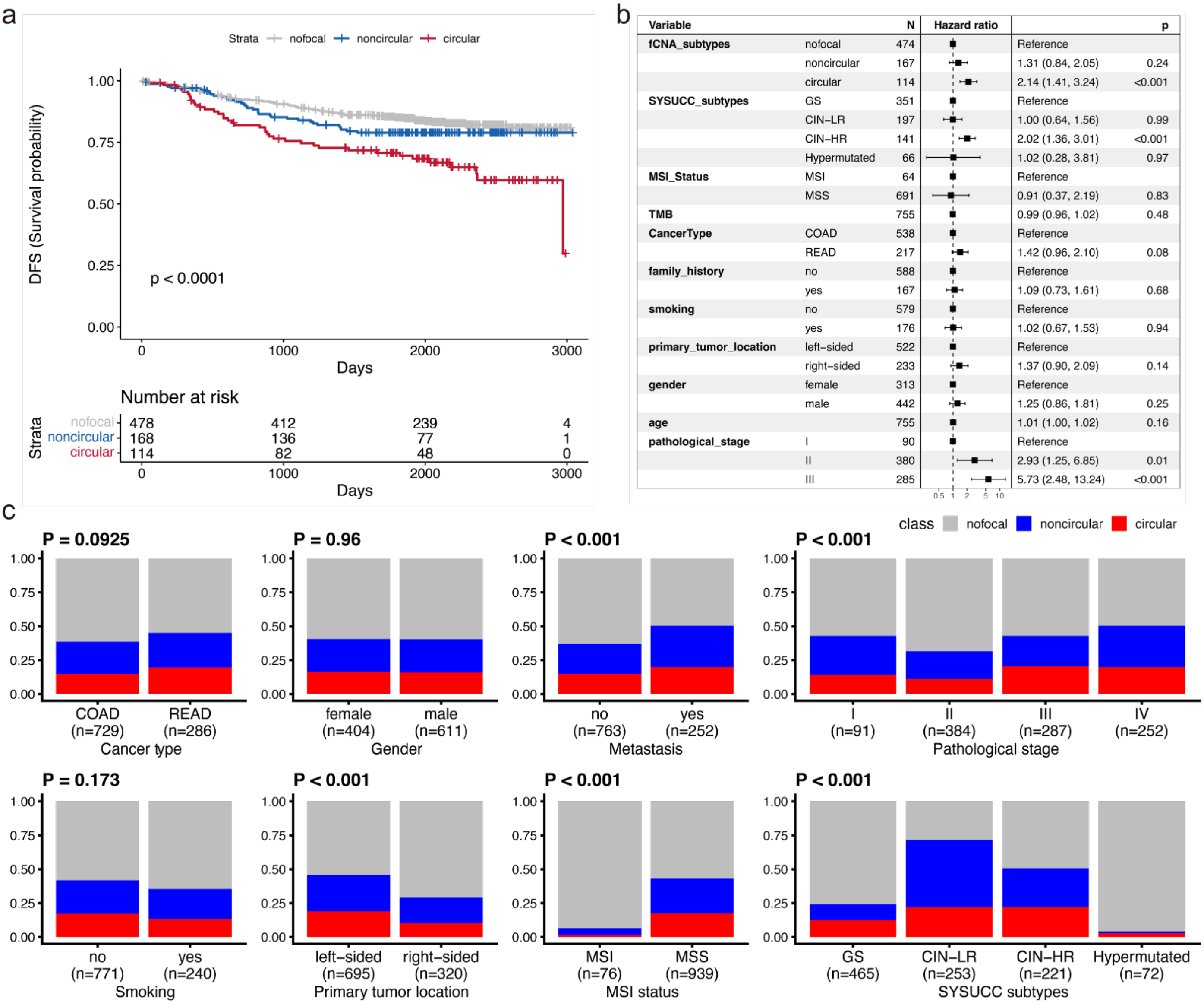

Supplementary Figure 6 | Association of focal amplification subtypes with disease-free survival and clinical categorical features.

a, Disease-free survival curve comparison between different focal amplification subtypes. **b**, Forest plots of multivariable disease-free survival Cox regression analysis for focal amplification subtypes with reported SYSUCC subtypes and other common clinical variables as confounding factors. **c**, Association analysis between focal amplification subtypes and annotated categorical variables. *P* values yielded by Fisher test are shown.

Supplementary Figure 7

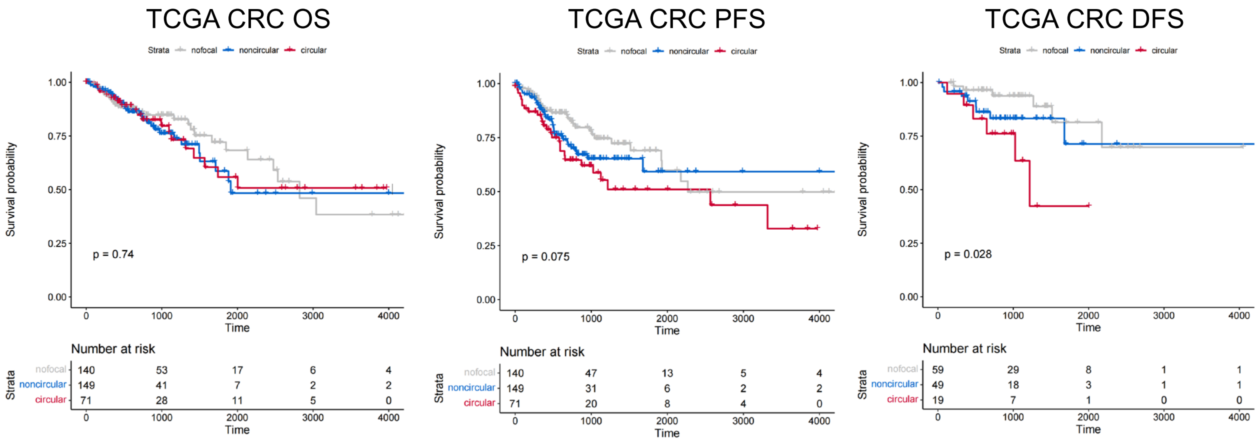

Supplementary Figure 7 | Kaplan-Meier overall survival (OS), progression-free survival (PFS), and disease-free survival (DFS) curve comparisons between different focal amplification subtypes in TCGA colorectal cancer.

Supplementary Figure 8

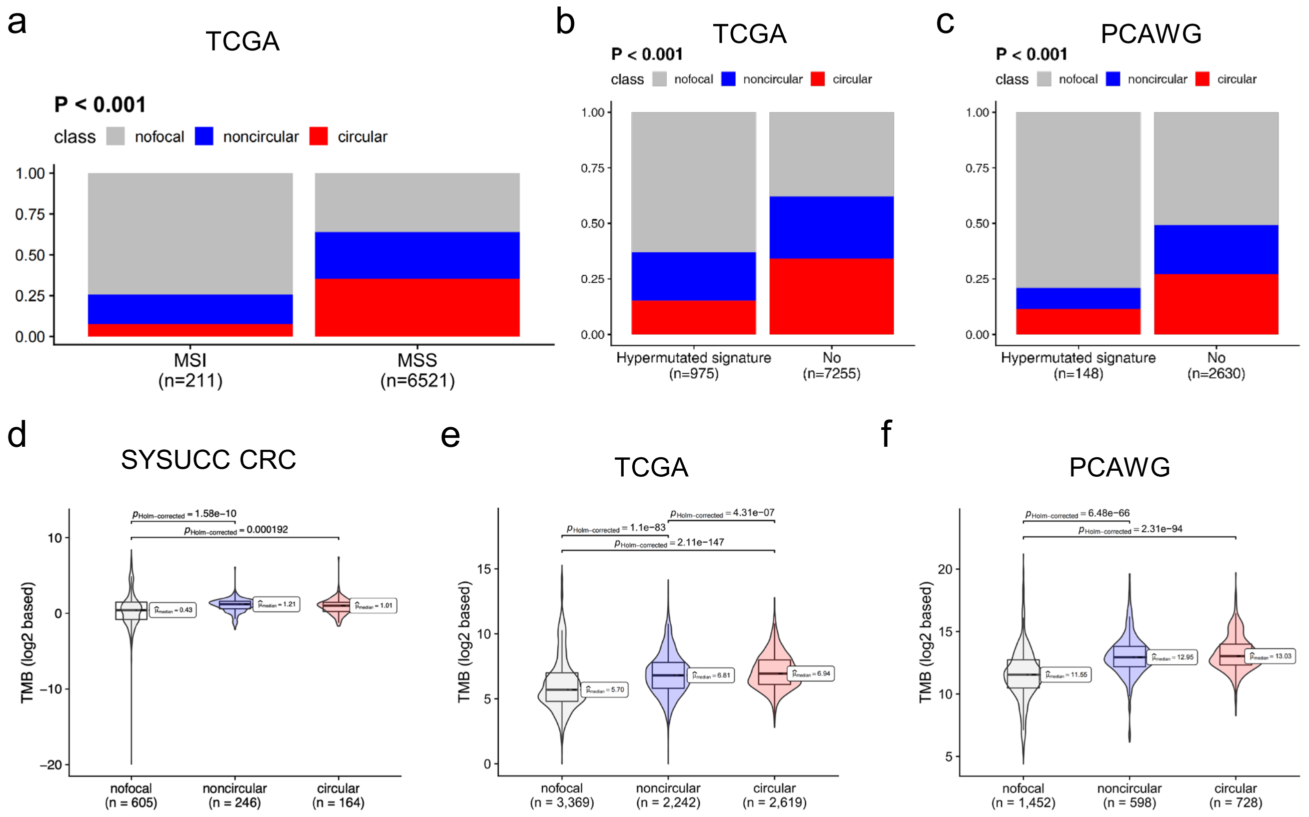

Supplementary Figure 8 | Analysis of MSI status and tumor mutation burden (TMB). **a**, Circular amplification enriches in TCGA microsatellite stable (MSS) tumors. This was validated in both (**b**) TCGA and (**c**) PCAWG with hypermutated signature, which was determined by checking if a tumor has any contribution from dMMR (deficiency in mismatch repair) and POLE/POLD1 related mutational signatures. **d**, Comparison of TMB between different focal amplification subtypes in the SYSUCC CRC cohort. **e**, Comparison of TMB between different focal amplification subtypes in the TCGA database. **f**, Comparison of TMB between different focal amplification subtypes in the PCAWG database.

Supplementary Figure 9

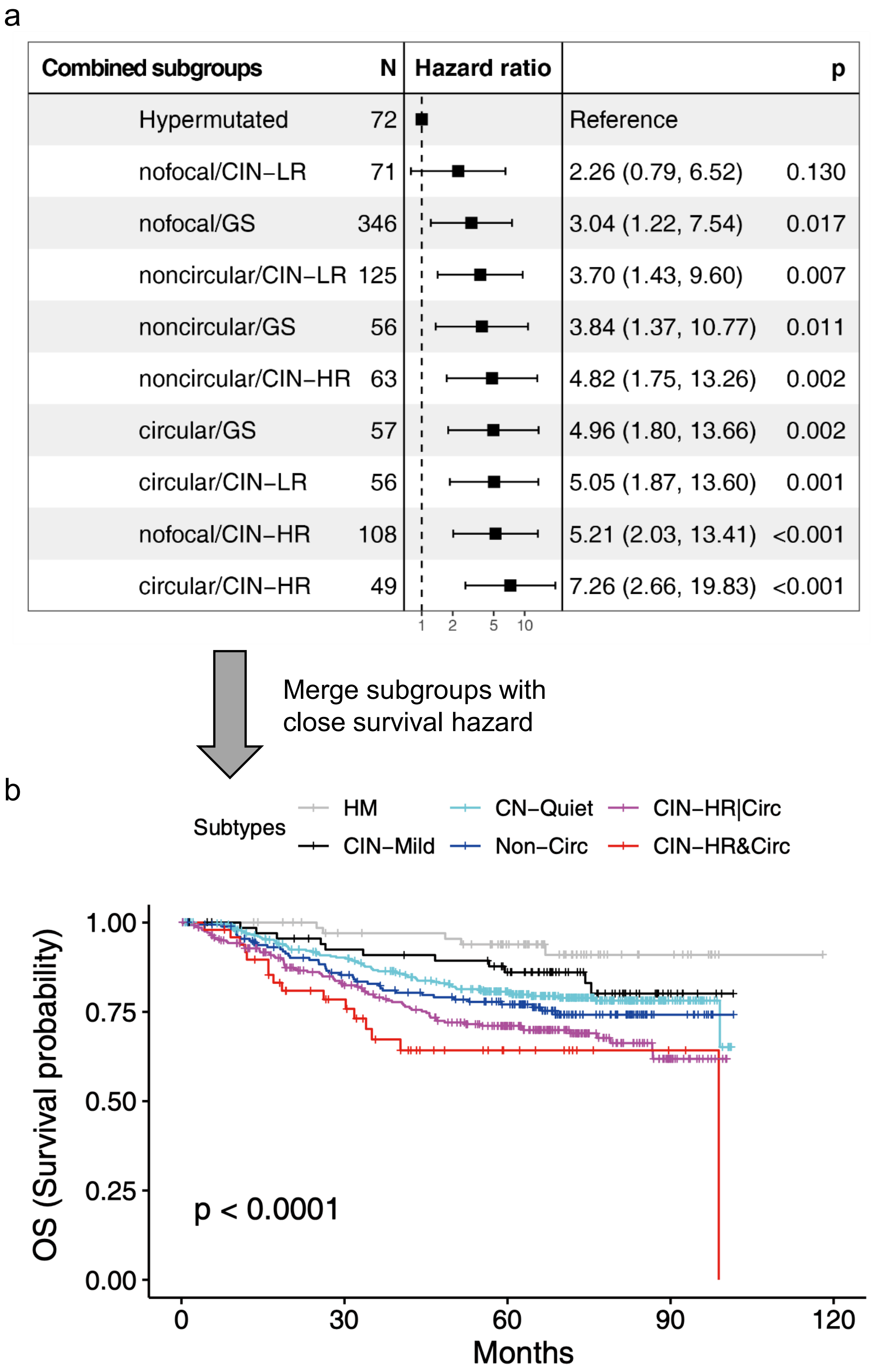

Supplementary Figure 9 | Creation of new CRC genomic subtypes.
a, Overall survival forest plot and hazard ratios of combined SYSUCC subgroups generated by label combination of existing SYSUCC subtypes and focal amplification subtypes. **b**, Kaplan-Meier overall survival curve comparison between new SYSUCC CRC genomic subtypes.

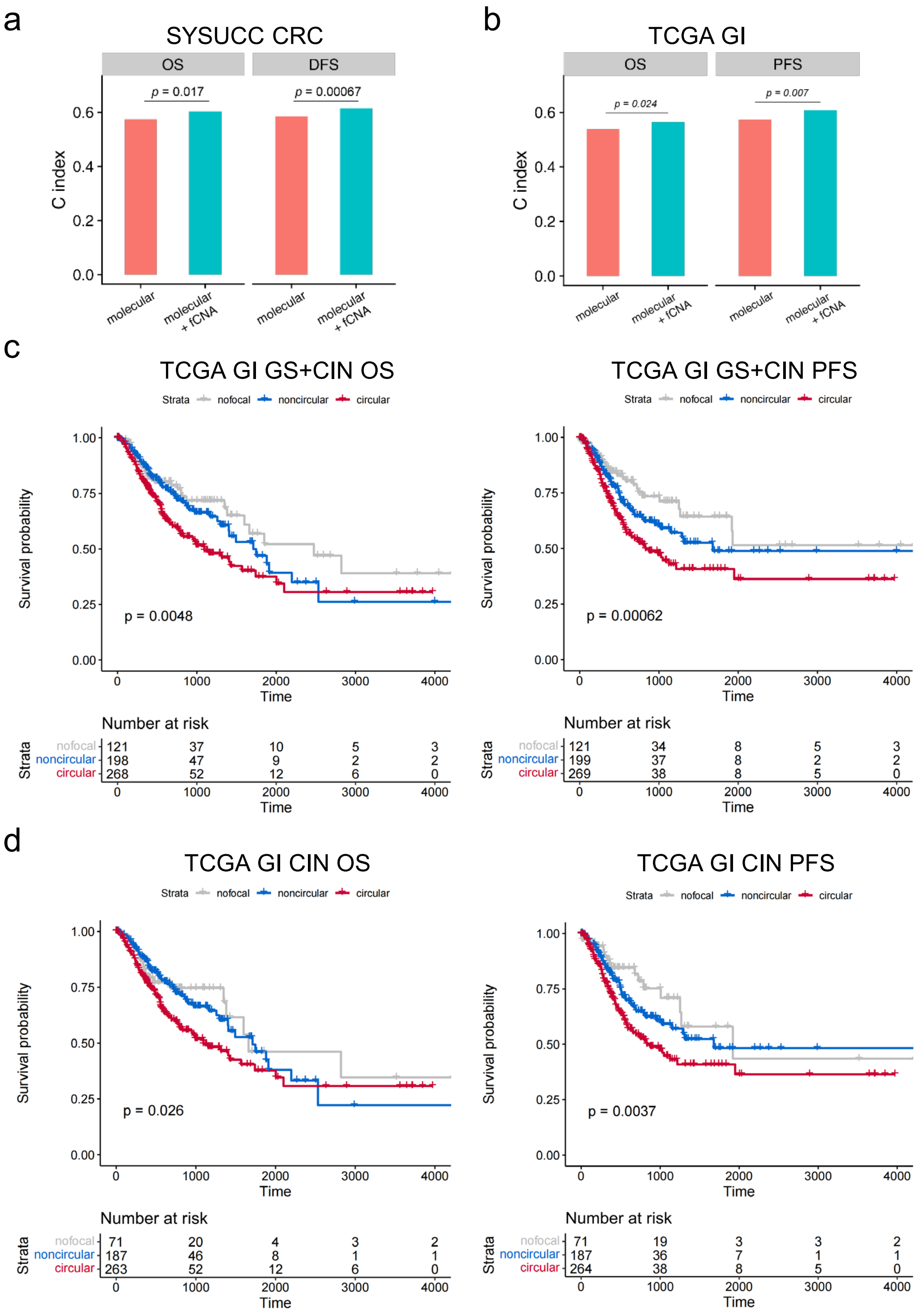

Supplementary Figure 10 | Focal amplification typing refines cancer genomic subtypes by stratifying chromosomal instability (CIN) tumors.

a, Focal amplification typing statistically improves the survival stratification (C index) in the SYSUCC CRC cohort. The *P* values were generated by ANOVA on two compared Cox regression models. **b**, Focal amplification typing statistically improves the survival stratification (C index) in TCGA gastrointestinal (GI) cancer. The *P* value were generated by ANOVA on two compared Cox regression models. **c**, Kaplan-Meier overall survival (OS) and progression free survival (PFS) curve comparisons between different focal amplification subtypes in genome stable (GS) and chromosomal instability (CIN) TCGA gastrointestinal cancer. **d**, Kaplan-Meier overall survival (OS) and progression free (PFS) survival curve comparisons between different focal amplification subtypes in TCGA CIN gastrointestinal cancer.

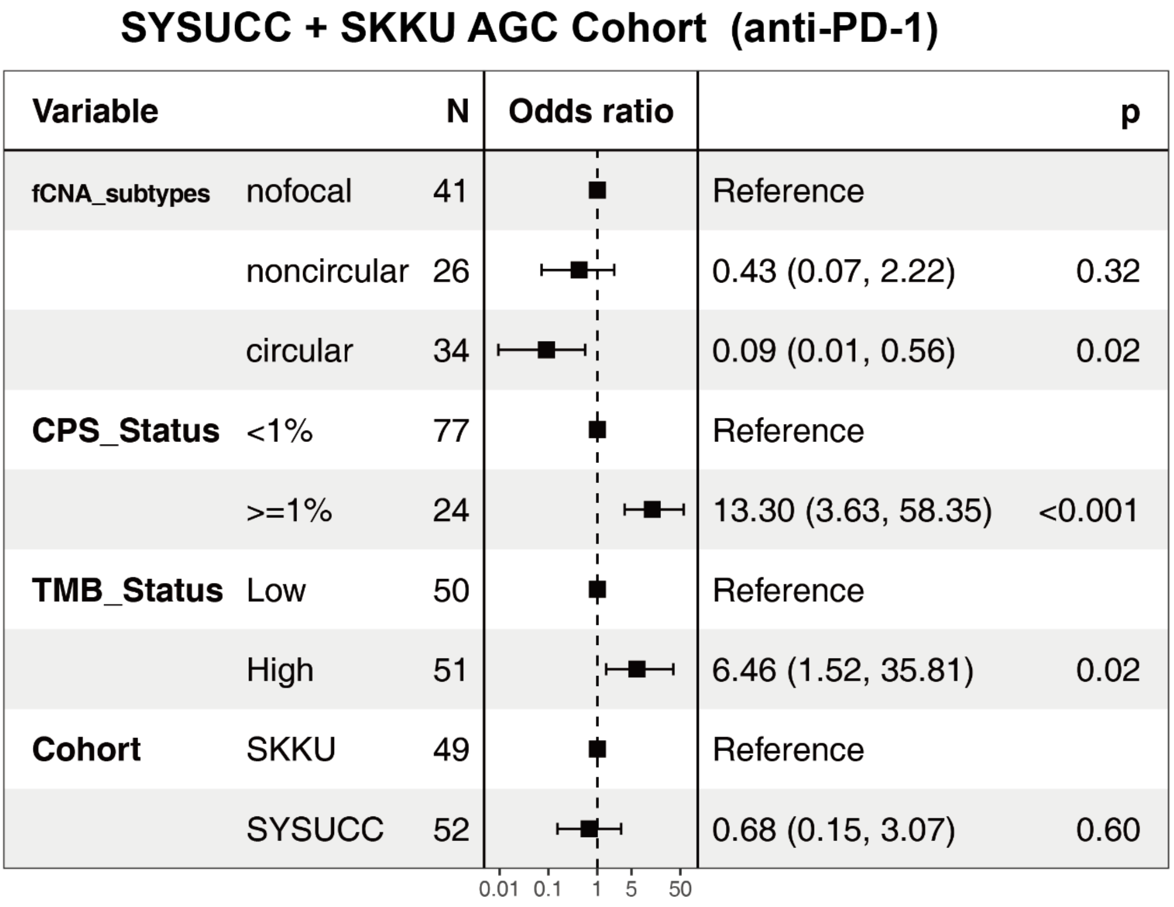

Supplementary Figure 11 | Forest plot showing circular amplification predicts anti-PD-1 nonresponse in advanced gastric cancer with a multivariable logistic regression model.

Two anti-PD1 clinical cohorts, including SYSUCC AGC and SKKU AGC, had response data, so they were combined here. Abbr.: CPS_Status, status of PDL1 combined positive score; TMB_Status, status of tumor mutation burden.

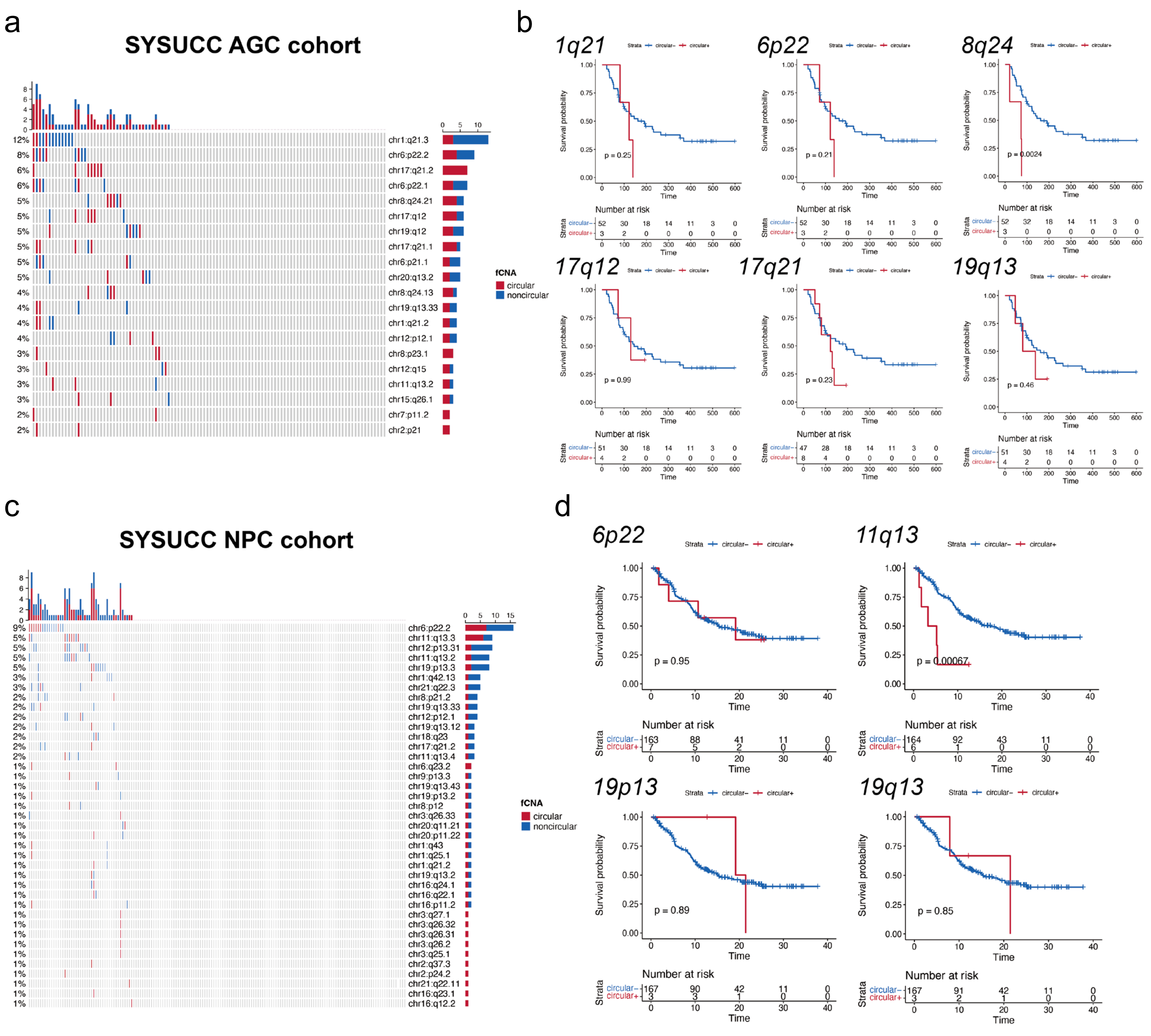

Supplementary Figure 12 | Cytoband-level analysis of focal amplifications in two anti-PD1 cancer cohorts.

a, Oncoplot depicting focal amplifications in genome cytobands with >1 circular-amplified patients in the SYSUCC AGC cohort. **b**, Kaplan-Meier overall survival comparison among cancer patients stratified by circular amplification status of specific cytobands in the SYSUCC AGC cohort. **c**, Oncoplot depicting focal amplifications in genome cytobands in the SYSUCC NPC cohort. **d**, Kaplan-Meier overall survival comparison among cancer patients stratified by circular amplification status of specific cytobands in the SYSUCC NPC cohort. Log-rank test *P* values are shown.

Supplementary Figure 13

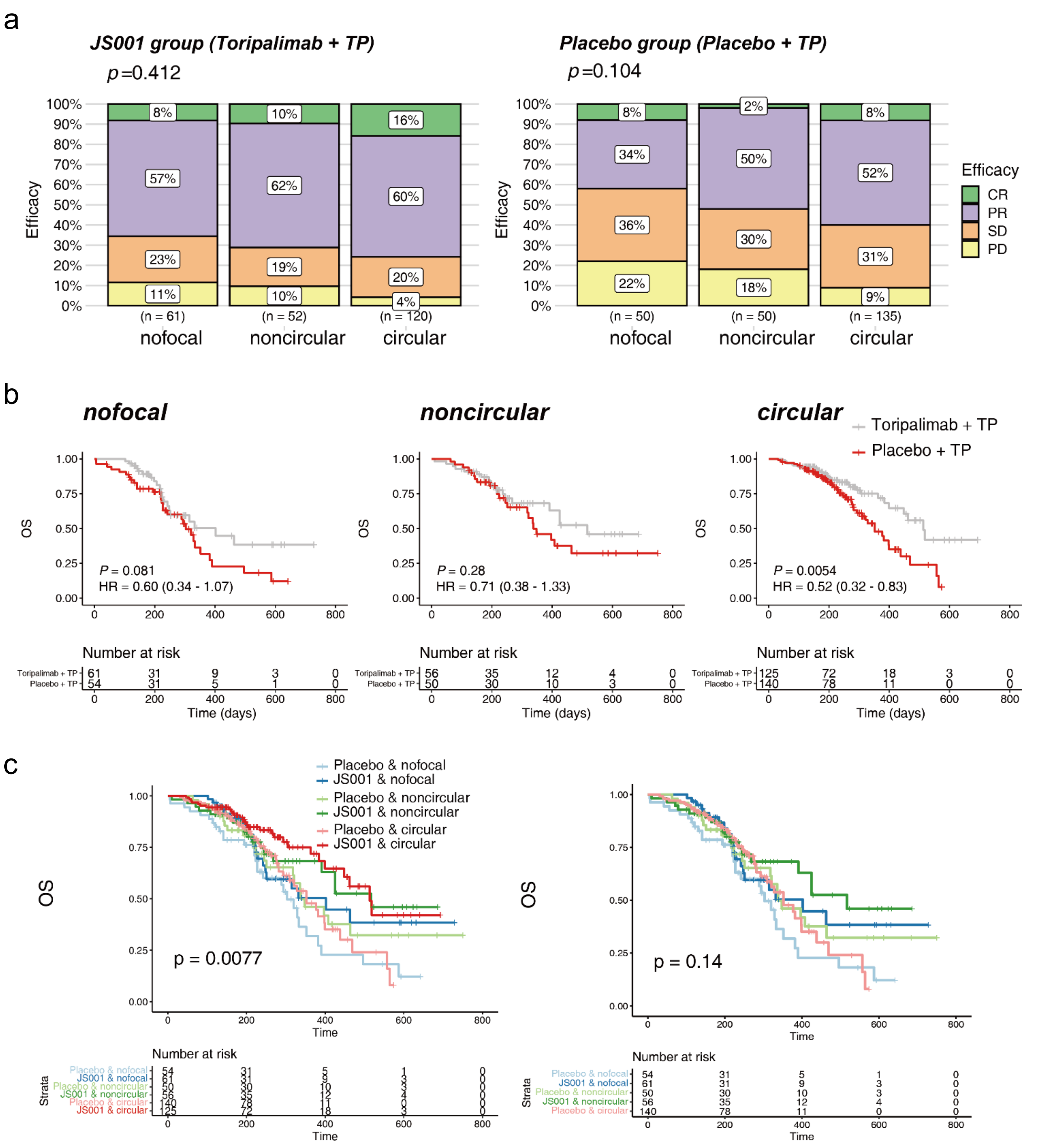

Supplementary Figure 13 | Circular amplification predicts good prognosis of combination immunotherapy in advanced ESCC (JUPITER-06 cohort).
a, Subtype circular tends to have more CR+PR rate and less PD rate in both JS001 group (treat with toripalimab + paclitaxel and cisplatin (TP)) and Placebo group (treat with placebo + TP). **b**, Kaplan-Meier overall survival comparison between patients treated with toripalimab + TP and patients treated with placebo + TP faceted by different focal amplification subtypes. Log-rank test *P* value and hazard ratio (95% confidence interval) are shown. **c**, Circular amplified patients treated with toripalimab + TP achieve the best overall survival. Kaplan-Meier overall survival (OS) curve comparison between different subgroups (generated by combining treatment groups and focal amplification subtypes) (left panel). Log-rank test *P* value is shown. A specific subgroup JS001 & circular is removed to regenerate Kaplan-Meier overall survival curve and corresponding log-rank test *P* value shown in the right panel.

Supplementary Figure 14

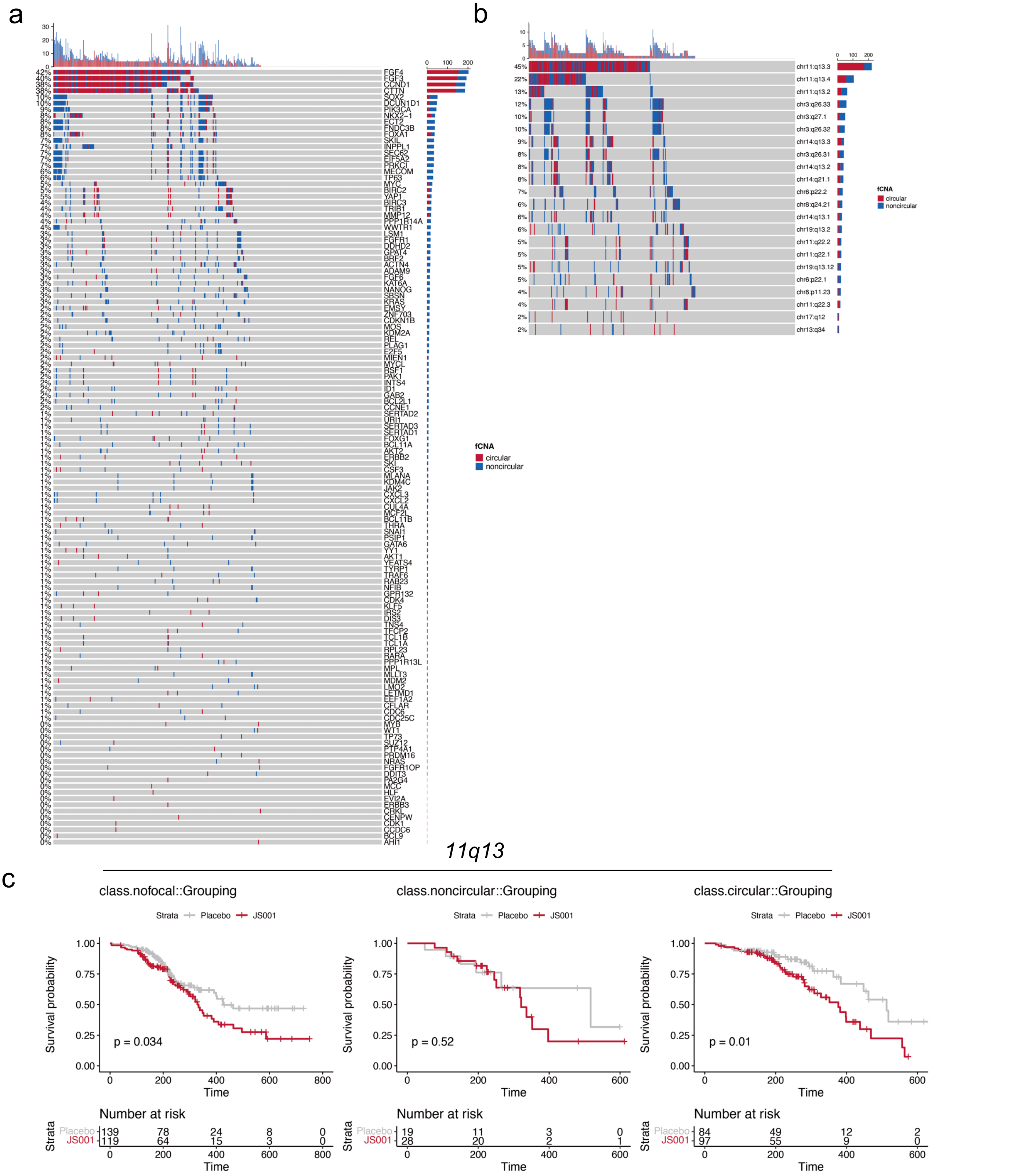

Supplementary Figure 14 | Gene-level and cytoband-level analysis in JUPITER-06 cohort. **a**, Oncoplot depicting gene-level focal amplifications. **b**, Oncoplot depicting cytoband-level focal amplifications. Here, only genome cytobands with >5 circular-amplified patients are shown. **c**, Kaplan-Meier overall survival comparison between patients treated with toripalimab + TP (JS001 group) and patients treated with placebo + TP (Placebo group) faceted by different focal amplification subtypes in 11q13. Log-rank test *P* values are shown.

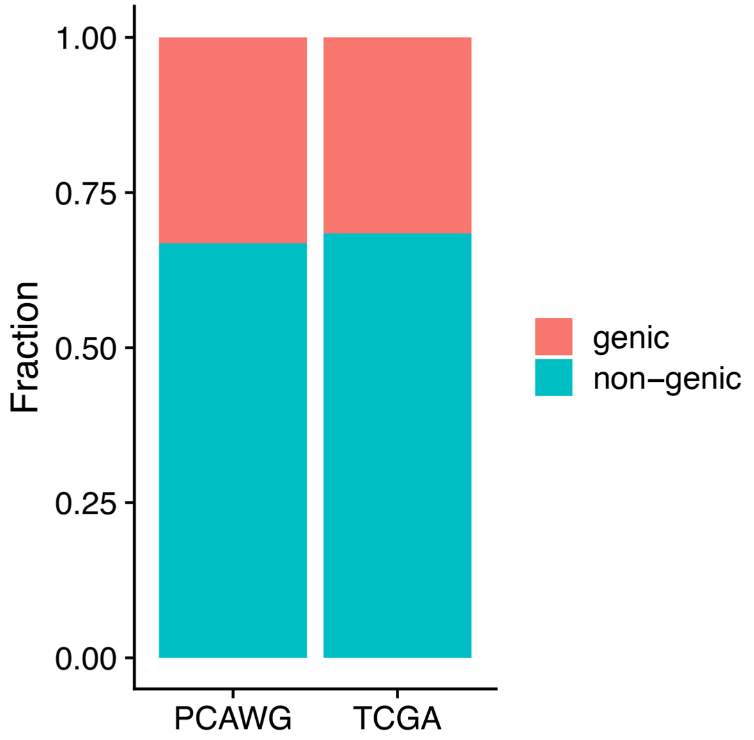

Supplementary Figure 15 | Non-genic genomic elements are abundant in ecDNAs, highlighting the presence of various structural features beyond coding regions.

Supplementary method for manuscript:

Machine learning-based extrachromosomal DNA identification in large-scale cohorts reveals its clinical implications in cancer

Shixiang Wang[1,#], Chen-Yi Wu[1,#], Ming-Ming He[1,#], Jia-Xin Yong[1,#], Yan-Xing Chen[1], Li-Mei Qian[1], Jin-Ling Zhang[1], Zhao-Lei Zeng[1], Rui-Hua Xu[1,2,*], Feng Wang[1,*], Qi Zhao[1,*]

Affiliations of authors:

[1] State Key Laboratory of Oncology in South China, Guangdong Key Laboratory of Nasopharyngeal Carcinoma Diagnosis and Therapy, Guangdong Provincial Clinical Research Center for Cancer, Sun Yat-sen University Cancer Center, Guangzhou 510060, P. R. China.

[2] Research Unit of Precision Diagnosis and Treatment for Gastrointestinal Cancer, Chinese Academy of Medical Sciences, Guangzhou, 510060, P. R. China.

# These authors contributed equally.

* These authors jointly supervised this work.

Correspondence: Qi Zhao, e-mail: zhaoqi@sysucc.org.cn; Feng Wang, e-mail: wangfeng@sysucc.org.cn; Rui-Hua Xu, e-mail: xurh@sysucc.org.cn;

Contents:

- Introduction
- GCAP modeling for ecDNA amplification identification
- GCAP implementation using R
- References

Introduction

This supplementary method file provides a detailed account of GCAP (Gene-level Circular Amplicon Prediction), an innovative approach designed to detect extrachromosomal DNA (ecDNA) using whole-exome sequencing (WES) data and absolute copy number profiles. Instead of delving into the background, performance, and validation aspects of GCAP, our focus remains on the practical aspects: describing the data collection and preprocessing methods, outlining the modeling process, and presenting the implementation framework achieved through R packages. Finally, we provide a concise guide on effectively employing the developed R packages.

GCAP modeling for ecDNA amplification identification

The modeling procedure is delineated through the following five sequential steps, with an overarching framework overview depicted in Supplementary Figure 16.

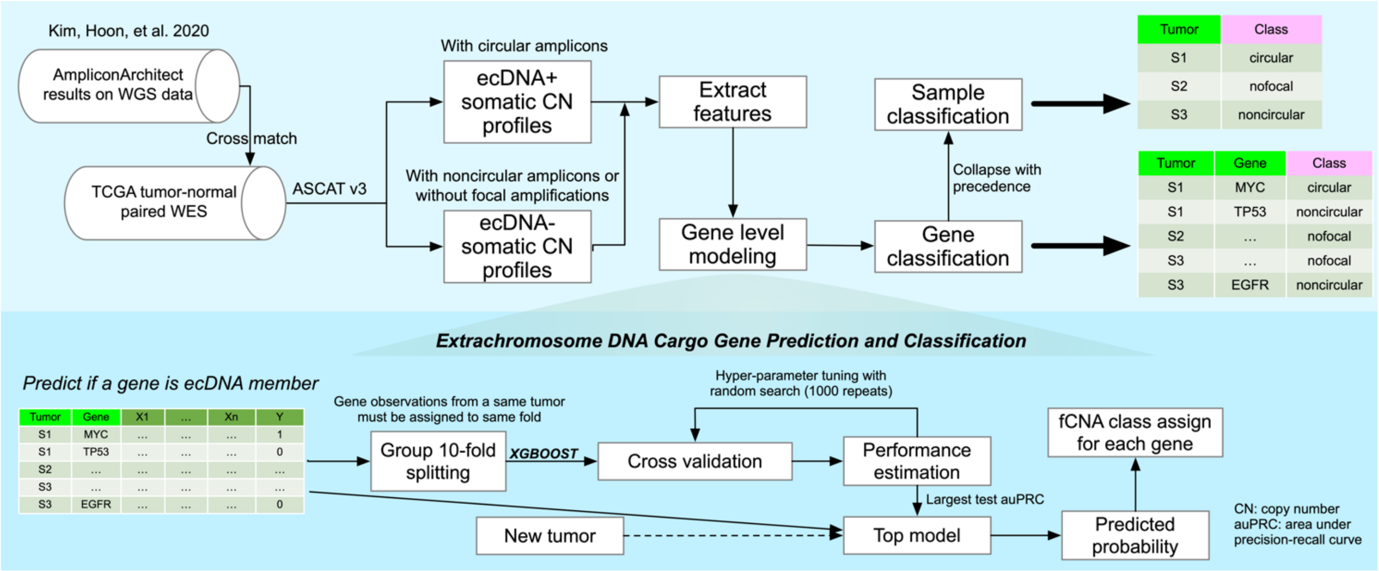

Supplementary Figure 16. Extrachromosomal DNA cargo gene prediction modeling and sample classification workflow.

Step 1. Data collection and preparation

Kim *et al.*[1] processed whole-genome sequencing (WGS) data from 3,212 tumors sourced from PCAWG and TCGA projects using the AmpliconArchitect[2] software. Each tumor possessing amplicons was

categorized into four primary classes with a hierarchical order: 'Circular' (ecDNA), 'BFB' (breakage-fusion-bridge), 'HR' (heavily-rearranged), and 'Linear' (linear amplification). Here, 'precedence' implies that if a tumor exhibits both 'Circular' and 'Linear' amplicons, it is labeled as a 'Circular' tumor. Tumors lacking any amplicons were classified as 'No-fSCNA'. In our study, to streamline the focus on ecDNA prediction modeling, we categorized 'Circular' as 'ecDNA+' and grouped all others as 'ecDNA-'. Furthermore, for enhanced analysis of the data, we also classified 'Circular' as circular amplification, amalgamated 'BFB', 'HR', and 'Linear' into noncircular amplification, and designated the remaining cases as 'nofocal'.

We gathered the AmpliconArchitect result classes of 3,212 tumors as reported by Kim *et al.*[1] and subsequently categorized these classes into either 'ecDNA+' or 'ecDNA-'. We excluded tumors without TCGA identifiers (those with sample barcodes not beginning with 'TCGA'). We retrieved the TCGA identifiers and employed them to access the GDC data portal[3] (https://gdc.cancer.gov/) to identify matched TCGA patients with tumor-normal paired WES data. Given the following considerations: 1) the prevalence of ecDNA-negative tumors; 2) most of gene regions in ecDNA+ tumors are ecDNA-negative; 3) AmpliconArchitect might categorize certain ecDNA+ structures as intricate and non-cyclic if breakpoints are overlooked[4]; 4) the occurrence of BFB cycles can lead to the development of ecDNA, complicating the differentiation between these two modes of amplification[5]; and 5) the gene-level data modeling is resource-intensive, which necessitates stringent control at both gene and sample levels – we retained a total of 326 matched ecDNA+ tumors while also randomly selecting 30 ecDNA- tumors exhibiting 'Linear' amplification (representing non-ecDNA focal amplification) and 30 'No-fSCNA' (representing no focal amplification) ecDNA- tumors (by the R function *sample_n* in dplyr package with random seed ‘2021’).

We obtained the TCGA raw WES bam files (aligned by TCGA team with hg38 as reference) for the modeling process using the GDC data transfer tool, gdc-client (https://gdc.cancer.gov/access-data/gdc-data-transfer-tool). Additionally, we acquired clinical annotations of TCGA patients from UCSC Xena[6] through the UCSCXenaShiny[7] platform.

Step 2. Generation of predictive features and response variable for modeling

We employed ASCAT v3[8] to preprocess the TCGA raw whole-exome sequencing (WES) data using hg38 as the reference genome. This preprocessing yielded essential data, including allele-specific copy number

profiles (total_cn and minor_cn), as well as estimations of tumor purity and ploidy. Detailed information about this process can be found in the 'Allele specific copy number calling and feature extraction' section of the Methods in the formal manuscript.

To facilitate copy number calling execution in HPC platform and ensure analysis reproducibility, we utilized a specific version of ASCAT (https://github.com/ShixiangWang/ascat/tree/v3.0). Subsequently, we calculated various genomic alteration measures, such as pLOH (percentage of the genome with LOH), AScore (aneuploidy score), and cna_burden (copy number alteration burden), using Sigminer[9], taking the allele-specific copy number profiles as input. Consequently, we obtained seven crucial features: total_cn (total copy number), minor_cn (copy number of the minor allele), purity, ploidy, pLOH, AScore, and cna_burden.

Human gene annotation data were sourced from GENCODE (gencode.v38.annotation.gtf.gz, https://www.gencodegenes.org/), enabling the extraction of information such as gene position and ENSEMBL gene identifier for all protein-coding genes. With the data, we collapsed and unified the seven features at gene-level (only genes in autosome were considered). AmpliconArchitect amplicon classes for specific regions of 3,212 tumors, as detailed in the supplementary file by Kim *et al.*[1], were gathered. After data cleaning, we obtained amplicon classes ('Circular', 'BFB', 'HR', and 'Linear') for each genomic region and filtered out non-modeling samples. Considering Kim *et al.*'s use of hg19 as a reference for AmpliconArchitect, we obtained the human gene annotation data for hg19 from GENCODE (gencode.v38lift37.annotation.gtf.gz). From this source, we extracted pertinent details for all protein-coding genes. Through the overlap analysis of gene and amplicon regions, we generated the response variable for our modeling endeavor, namely, whether an observed gene serves as an ecDNA cargo gene (where a gene region intersects with a 'Circular' amplicon in a sample). Furthermore, we calculated the frequency of the four amplicon classes for each gene, taking into account 1,000 times the count of samples where an amplicon overlaps a gene, divided by 3,212 (the multiplication by 1,000 was employed to establish a suitable value range).

Through the integration of the aforementioned seven features, along with the frequency of four amplicon classes on a gene-level basis for each sample, we derived a fundamental feature matrix comprising 7,279,221 observations and 11 predictive features for modeling. Of which, only 0.35% (25,724

observations) are ecDNA positive. Furthermore, to assess whether incorporating additional features, such as patient clinical information, copy number signatures[10], and cancer types, could enhance model performance, we constructed two additional feature sets: one with 32 features (11 features + patient age + patient sex + 19 copy number signatures), and another with 56 features (32 features + 24 cancer types (386 tumors in total)). Categorical variables (sex and cancer types) were encoded using one-hot encoding.

Step 3. Modeling and hyper-parameter searching

To address imbalanced modeling data and prevent information leakage across the train-test division[11], we adopted the area under the precision-recall curve (auPRC) as our evaluation metric. Additionally, we implemented a group k-fold strategy, with k being 10 by default. However, we also utilized k values of 3, 5, 10, and 20 for specific purposes, such as comparison.

To elaborate, all cancer patients earmarked for modeling were sorted based on the number of ecDNA cargo genes, and subsequent random sampling was conducted. This resulted in a distribution of 90% of samples for the training fold and 10% for the testing fold. Furthermore, all gene-level observations linked to each cancer patient were appropriately assigned to the respective fold. In other words, observations from the same cancer patient were allocated exclusively to either the training or testing fold, but not both.

In line with these arrangements, we fine-tuned the XGBOOST model using 10-fold cross-validation data, encompassing a search space of 1,000 hyperparameters drawn from a random selection process.

Drawing guidance from the XGBOOST documentation (https://xgboost.readthedocs.io/en/stable/tutorials/param_tuning.html) and based on our preliminary investigations, we conducted hyperparameter tuning through a random search approach with R code provided below.

```
N <- 1000
search_space <- data.frame(
  eta = sample(c(0.01, 0.1, 0.3), N, replace = TRUE, prob = c(0.2, 0.6, 0.2)),
  max_delta_step = sample(c(0, 1, 10), N, replace = TRUE, prob = c(0.2, 0.6, 0.2)),
  max_depth = sample(2:6, N, replace = TRUE),
```

```
  min_child_weight = sample(c(1, 2, 5, 10, 20, 100), N, replace = TRUE, prob = c(0.1, 0.3, 0.2,
0.2, 0.1, 0.1)),
  alpha = sample(c(0, 0.5, 1), N, replace = TRUE, prob = c(0.4, 0.3, 0.3)),
  lambda = sample(c(1, 0.5, 0), N, replace = TRUE, prob = c(0.4, 0.3, 0.3)),
  gamma = sample(c(0, 1, 10), N, replace = TRUE, prob = c(0.2, 0.4, 0.4)),
  subsample = sample(c(0.5, 0.6, 0.7, 0.8, 0.9), N, replace = TRUE, prob = rep(0.2, 5)),
  colsample_bytree = sample(c(0.6, 0.7, 0.8, 0.9, 1), N, replace = TRUE, prob = rep(0.2, 5))
)
```

Step 4. Final model determination

The ultimate set of hyperparameters was selected from the cross-validation model that exhibited the highest auPRC (XGB11 - eta: 0.1, max_delta_step: 0, max_depth: 4, min_child_weight: 1, alpha: 0, lambda: 1, gamma: 10, subsample: 0.6, colsample_bytree: 1, objective: logistic, eval_metric: aucpr). These refined hyperparameters were subsequently employed across the entire modeling dataset to derive the final XGBOOST model for practical utilization.

Step 5. Focal amplification-based gene and sample classification

To distinguish between extrachromosomal DNA amplification and chromosomal DNA amplification, the prediction probabilities of ecDNA cargo gene (obtained from the XGBOOST model prediction) were utilized to classify various focal amplification subtypes according to the following guidelines:

As a prerequisite, focal amplification should lead to at least a four-copy increase beyond tumor ploidy, in line with the previously outlined criteria by Kim *et al.*[1]

Subsequently, the gene classification process unfolded as follows:

1. A gene was assigned to the "nofocal" category (indicating no focal amplification detected) if its total copy number was less than tumor ploidy + 4 copies.

2. A gene was categorized as "circular" (indicating extrachromosomal DNA amplification) if the associated probability exceeded 0.5.

3. A gene was designated as "noncircular" (indicating chromosomal DNA amplification) if the

probability was less than 0.5.

Based on these gene classifications, the categorization of a tumor was established by identifying the predominant focal amplification type within that tumor. To enhance the precision of classification, as a default criterion, a 'circular' tumor was required to possess at least one predicted ecDNA cargo gene with a probability exceeding 0.6.

The classification prioritized circular amplification, as per the approach described by Kim *et al.*[1] For instance, a tumor showcasing both circular and noncircular amplifications would be categorized as "circular," while a tumor with solely noncircular amplification would be labeled as "noncircular." In cases where no focal amplifications were evident (i.e., all investigated genes were assigned to the "nofocal" category), the tumor classification would be "nofocal."

The definitions of 'circular' and 'nofocal' remain consistent with those outlined in Kim *et al.*'s study. Unlike the approach taken by Kim *et al.*, we did not refine chromosomal amplicons (i.e., 'noncircular') stemming from breakage-fusion-bridge (BFB) and heavily-rearranged (HR) mechanisms. This was primarily due to the challenge of characterizing and distinguishing them from linear amplifications (Linear) based solely on WES data.

GCAP implementation using R

R package GCAP and command-line interfaces

We have encapsulated our constructed XGBOOST models and realized comprehensive end-to-end analysis workflows within an R package named GCAP. GCAP is accessible for academic use free of charge and can be obtained from https://github.com/ShixiangWang/gcap.

To optimize the usability of GCAP as a prototypical bioinformatics pipeline intended for operation within a Linux command line environment, we have designed two command-line interfaces (CLIs) using the R package GetoptLong (https://github.com/jokergoo/GetoptLong). These CLIs, named `gcap-bam.R` and `gcap-ascn.R`, are tailored for managing input data originating from tumor-normal paired BAM files and allele-specific copy number profile files, respectively. Additionally, we have developed a central command interface, `gcap`, which seamlessly integrates the functionality of the two CLIs, capitalizing on the capabilities introduced by the new feature in GetoptLong v1.1.0

(https://github.com/jokergoo/GetoptLong/issues/10; thanks to Zuguang Gu).

The fundamental operational framework of GCAP, encompassing the contributions of the CLIs, is illustrated in Supplementary Figure 17.

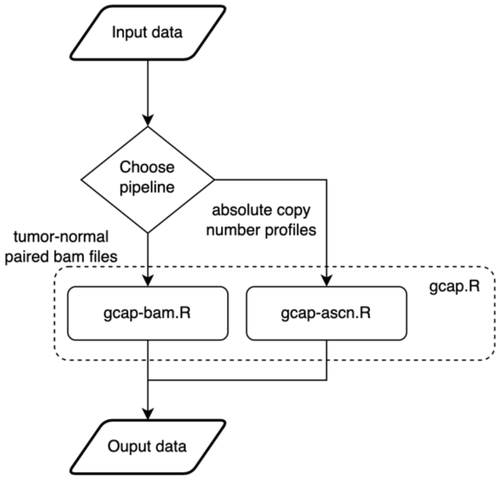

Supplementary Figure 17. Command line interfaces of GCAP.

The `gcap` interface is presented as follows:

```
$ gcap

gcap (v1.0.0)

Usage: gcap [command] [options]



Commands:

  bam     Run GCAP workflow with tumor-normal paired BAM files

  ascn    Run GCAP workflow with curated allele-specific copy number data
```

Module structure of R package GCAP

In a more detailed context, the functionality of the aforementioned two CLIs is underpinned by R functions, namely `gcap.workflow()` and `gcap.ASCNworkflow()`. These functions encompass both independent and shared functional modules, each dedicated to facilitating specific aspects of the key analysis procedures (Supplementary Figure 18).

These include:

➢ `gcap.runASCAT()`: run ASCAT v3 on tumor-normal pair WES data files to get allele-specific copy

number profiles.

- `gcap.runBuildflow()`: get data for prediction from ASCAT result files.
- `gcap.runASCNBuildflow()`: get data for prediction from absolute copy number data.
- `gcap.extractFeatures()`: extract sample and region level features.
- `gcap.collapse2Genes()`: generate unified gene-level feature data.
- `gcap.runPrediction()`: execute ecDNA cargo gene prediction.
- `gcap.runScoring()`: summarize prediction result into gene/sample-level classifications.

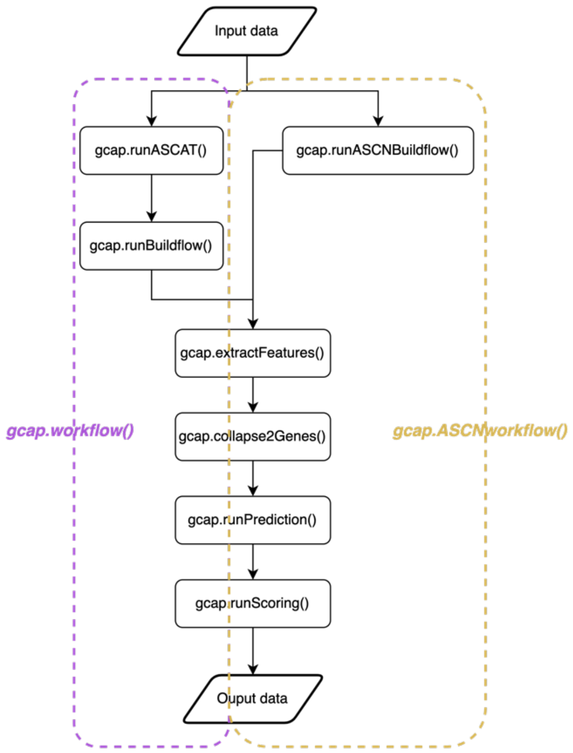

Supplementary Figure 18. GCAP workflows and their functional modules.

GCAP Docker container

For extended convenience in utilizing GCAP, we have additionally developed a Docker container for GCAP, accessible at https://github.com/ShixiangWang/gcap/pkgs/container/gcap. The timely updated Dockerfile is available at https://github.com/ShixiangWang/gcap/blob/master/Dockerfile.

Quickstart of using GCAP

Getting started with GCAP from allele-specific copy number data is straightforward. In this section, we provide a concise guide on installing the GCAP R package and utilizing it with pre-existing allele-specific copy number data.

1. Install R (https://cran.r-project.org/).
2. Install package remotes with R command (type it in R console):

```
install.packages("remotes")
```

3. Install package GCAP with R command:

```
remotes::install_github("ShixiangWang/gcap")
```

4. Now, run the analysis workflow with example data:

```
library(gcap)
data("ascn")
data <- ascn
rv <- gcap.ASCNworkflow(data, outdir = tempdir(), model = "XGB11")
```

If everything goes well, you would see the logging of analysis workflow indicating the result files, e.g.,

```
saving raw prediction result to /tmp/RtmpK3VbkP/gcap_3622cc08-3b43-11ee-8cdc-ac1f6bdc46e6_prediction_result.rds
Saving fCNA records and sample info to /tmp/RtmpK3VbkP/gcap_3622cc08-3b43-11ee-8cdc-ac1f6bdc46e6_fCNA_records.csv, /tmp/RtmpK3VbkP/gcap_3622cc08-3b43-11ee-8cdc-ac1f6bdc46e6_sample_info.csv
```

Also, you can view the result data object directly in R console:

```
> rv
======================
A <fCNA> object
  record: 213
    case: 2
      |__ (0) 56 noncircular
```

```
    |__ (2) 157 circular
======================
```

Furthermore, you have the flexibility to explore the result data object using the features supported by GCAP. For instance, you can obtain a gene summary for each focal amplification subtype as follows:

```
> rv$getGeneSummary()
             gene_id noncircular circular Total
  1: ENSG00000287908           0        1     1
  2: ENSG00000285982           0        1     1
  3: ENSG00000285133           0        1     1
  4: ENSG00000284188           0        1     1
  5: ENSG00000274443           0        1     1
 ---
209: ENSG00000066827           1        0     1
210: ENSG00000054796           1        0     1
211: ENSG00000042832           1        0     1
212: ENSG00000042813           1        0     1
213: ENSG00000008513           1        0     1
```

In conjunction with the R package GCAPutils (https://github.com/ShixiangWang/gcaputils), you gain the capability to comprehensively analyze and visualize the data. For instance, you can create a Kaplan-Meier curve plot to effectively compare survival differences across various focal amplification subtypes. An example (Supplementary Figure 19) is given below:

```
data("ascn")
data = ascn

# Create fake data
set.seed(1234)
```

```
data$sample = sample(LETTERS[1:10], nrow(data), replace = TRUE)
rv = gcap.ASCNworkflow(data, outdir = tempdir(), model = "XGB11")
rv$convertGeneID()

surv_data = data.frame(
  sample = rv$sample_summary$sample,
  time = 3000 * abs(rnorm(nrow(rv$sample_summary))),
  status = sample(c(0, 1), nrow(rv$sample_summary), replace = TRUE)
)
p = gcap.plotKMcurve(rv, surv_data)
p
```

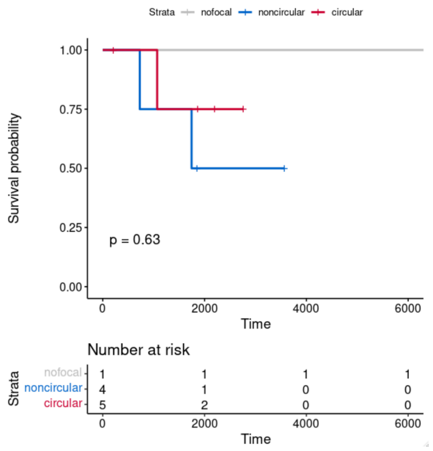

Supplementary Figure 19. An example K-M curve plot generated by GCAPutils package.

Package dependencies

We would like to express our sincere appreciation to all the developers of the dependent packages for gcap and gcaputils. Without the contributions of these exceptional individuals, our work would not have been possible.

```
ASCAT (>= 3.0.0),
xgboost,
data.table,
```

```
ggplot2,
plyr,
mltools,
Rcpp,
R6,
lgr,
PRROC,
GetoptLong,
circlize,
clusterProfiler,
ComplexHeatmap (>= 2.5.2),
cowplot,
ggrepel,
GenomicRanges,
survminer
```

More…

For further information about GCAP, please refer to the README of the R package project (https://github.com/ShixiangWang/gcap) and its corresponding documentation website (https://shixiangwang.github.io/gcap/).

If you have any feedback, please don't hesitate to file it through GitHub issues (https://github.com/ShixiangWang/gcap/issues) or contact us via email at wangsx1@sysucc.org.cn.

References

1. Kim, H. *et al.* Extrachromosomal DNA is associated with oncogene amplification and poor outcome across multiple cancers. *Nat. Genet.* **52**, 891–897 (2020).
2. Deshpande, V. *et al.* Exploring the landscape of focal amplifications in cancer using AmpliconArchitect. *Nat. Commun.* **10**, 392 (2019).
3. Liu, J. *et al.* An Integrated TCGA Pan-Cancer Clinical Data Resource to Drive High-Quality Survival Outcome Analytics. *Cell* **173**, 400-416.e11 (2018).

4. Lin, M. S. *et al. Transcriptional immune suppression and upregulation of double stranded DNA damage and repair repertoires in ecDNA-containing tumors*. http://biorxiv.org/lookup/doi/10.1101/2023.04.24.537925 (2023) doi:10.1101/2023.04.24.537925.
5. Yi, E., Chamorro González, R., Henssen, A. G. & Verhaak, R. G. W. Extrachromosomal DNA amplifications in cancer. *Nat. Rev. Genet.* 1–12 (2022) doi:10.1038/s41576-022-00521-5.
6. Goldman, M. *et al. The UCSC Xena platform for public and private cancer genomics data visualization and interpretation*. http://biorxiv.org/lookup/doi/10.1101/326470 (2018) doi:10.1101/326470.
7. Wang, S. *et al.* UCSCXenaShiny: an R/CRAN package for interactive analysis of UCSC Xena data. *Bioinformatics* **38**, 527–529 (2022).
8. Ross, E. M., Haase, K., Van Loo, P. & Markowetz, F. Allele-specific multi-sample copy number segmentation in ASCAT. *Bioinformatics* btaa538 (2020) doi:10.1093/bioinformatics/btaa538.
9. Wang, S. *et al.* Copy number signature analysis tool and its application in prostate cancer reveals distinct mutational processes and clinical outcomes. *PLOS Genet.* **17**, e1009557 (2021).
10. Steele, C. D. *et al.* Signatures of copy number alterations in human cancer. *Nature* (2022) doi:10.1038/s41586-022-04738-6.
11. Whalen, S., Schreiber, J., Noble, W. S. & Pollard, K. S. Navigating the pitfalls of applying machine learning in genomics. *Nat. Rev. Genet.* (2021) doi:10.1038/s41576-021-00434-9.

Supplementary FISH images for manuscript:

Machine learning-based extrachromosomal DNA identification in large-scale cohorts reveals its clinical implications in cancer

Shixiang Wang[1,#], Chen-Yi Wu[1,#], Ming-Ming He[1,#], Jia-Xin Yong[1,#], Yan-Xing Chen[1], Li-Mei Qian[1], Jin-Ling Zhang[1], Zhao-Lei Zeng[1], Rui-Hua Xu[1,2,*], Feng Wang[1,*], Qi Zhao[1,*]

Affiliations of authors:

[1] State Key Laboratory of Oncology in South China, Guangdong Key Laboratory of Nasopharyngeal Carcinoma Diagnosis and Therapy, Guangdong Provincial Clinical Research Center for Cancer, Sun Yat-sen University Cancer Center, Guangzhou 510060, P. R. China.

[2] Research Unit of Precision Diagnosis and Treatment for Gastrointestinal Cancer, Chinese Academy of Medical Sciences, Guangzhou, 510060, P. R. China.

# These authors contributed equally.

* These authors jointly supervised this work.

Correspondence: Qi Zhao, e-mail: zhaoqi@sysucc.org.cn; Feng Wang, e-mail: wangfeng@sysucc.org.cn; Rui-Hua Xu, e-mail: xurh@sysucc.org.cn;

Contents:

- Summary
- FISH images
- AmpliconArchitect results

Summary

This supplementary file comprises a collection of fluorescent in situ hybridization (FISH) images employed for the validation of GCAP. We performed FISH on 12 samples which were predicted as ecDNA positive by GCAP. These samples were harvested from 11 colorectal cancers patients, involving seven male and four female patients. All samples were predicted to exhibit ecDNA amplification for either *MYC* or *ERBB2*, except for one sample which seemed to present both genes amplification. For six samples with *ERBB2* amplification, supplementary validation results from AmpliconArchitect have also been provided.

Here, we employed scanning electron microscopy to comprehensively scan each tissue sample section. From each sample, we chose the most representative image illustrating the FISH results for individual gene probes. All raw image files are accessible to the public on Zenodo at https://zenodo.org/doi/10.5281/zenodo.7272630. It's worth noting that the majority of the images display relatively low signal-to-noise ratios. Nonetheless, upon zooming in and conducting a detailed manual inspection, it becomes evident that nearly all images display either diffuse gene amplification signals (**more confident**) and/or clustered gene amplification signals (**less confident**) highlighted within cyan boxes. This observation closely corresponds with the ecDNA amplification patterns detailed in our main manuscript, providing supplementary evidence that further reinforces the effectiveness of GCAP in clinical samples.

FISH images

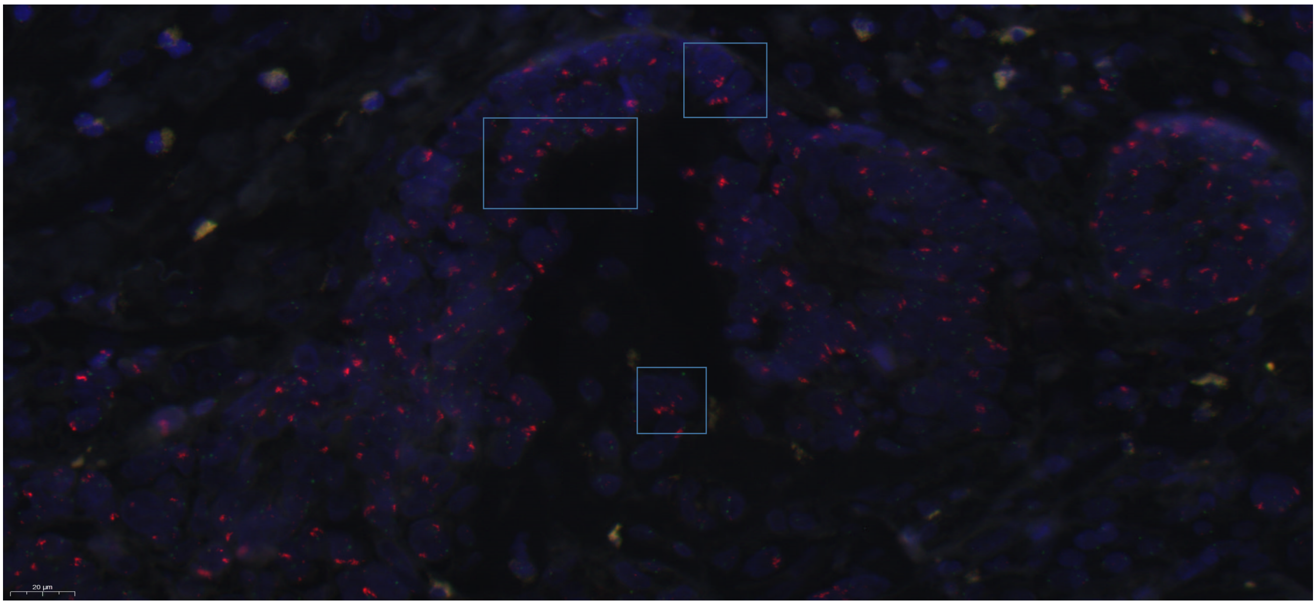

Supplementary Figure 20. CRC320, Male, *MYC* (63X).

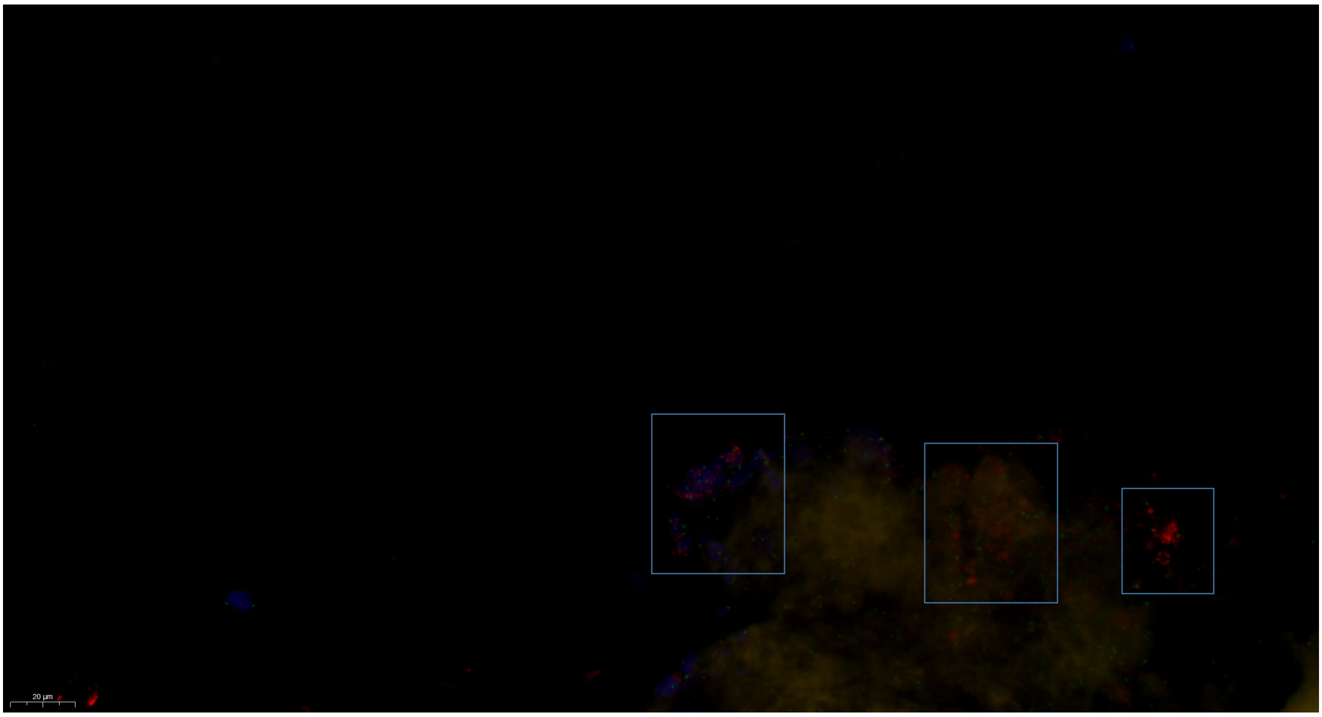

Supplementary Figure 21. CRC212, Female, *MYC* (63X).

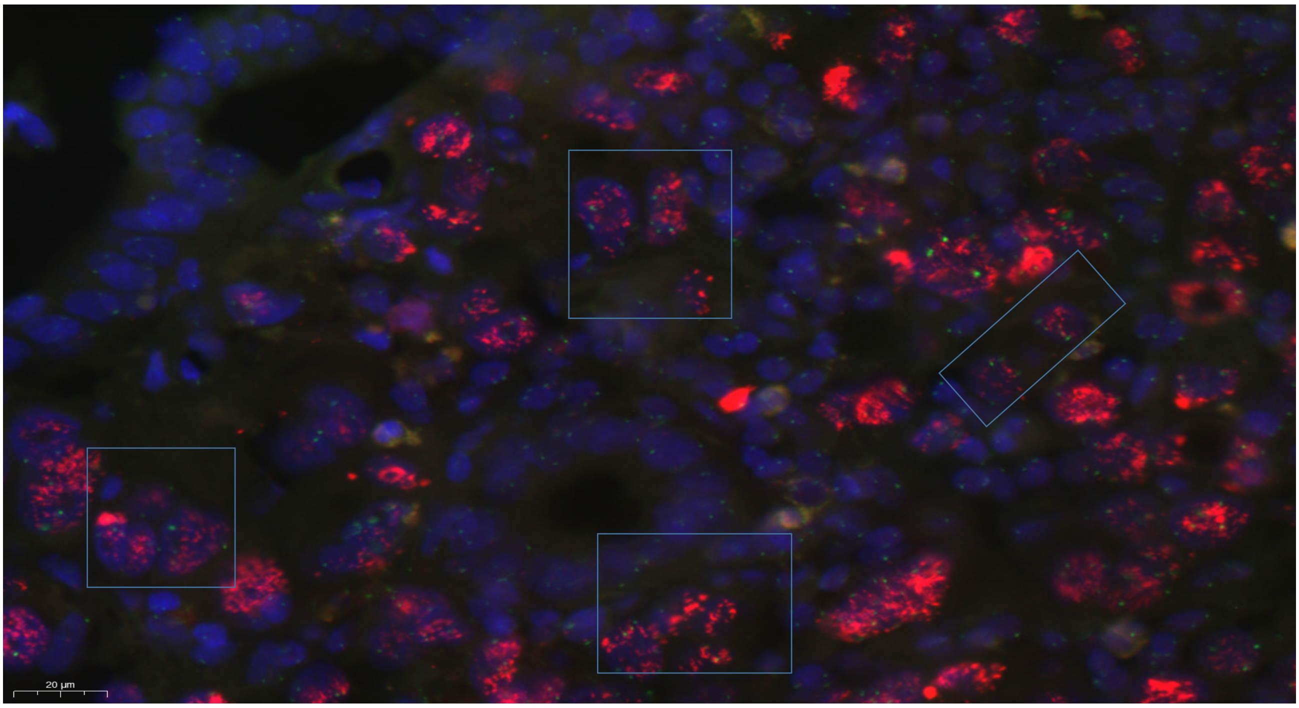

Supplementary Figure 22. CRC100, Male, *MYC* (63X).

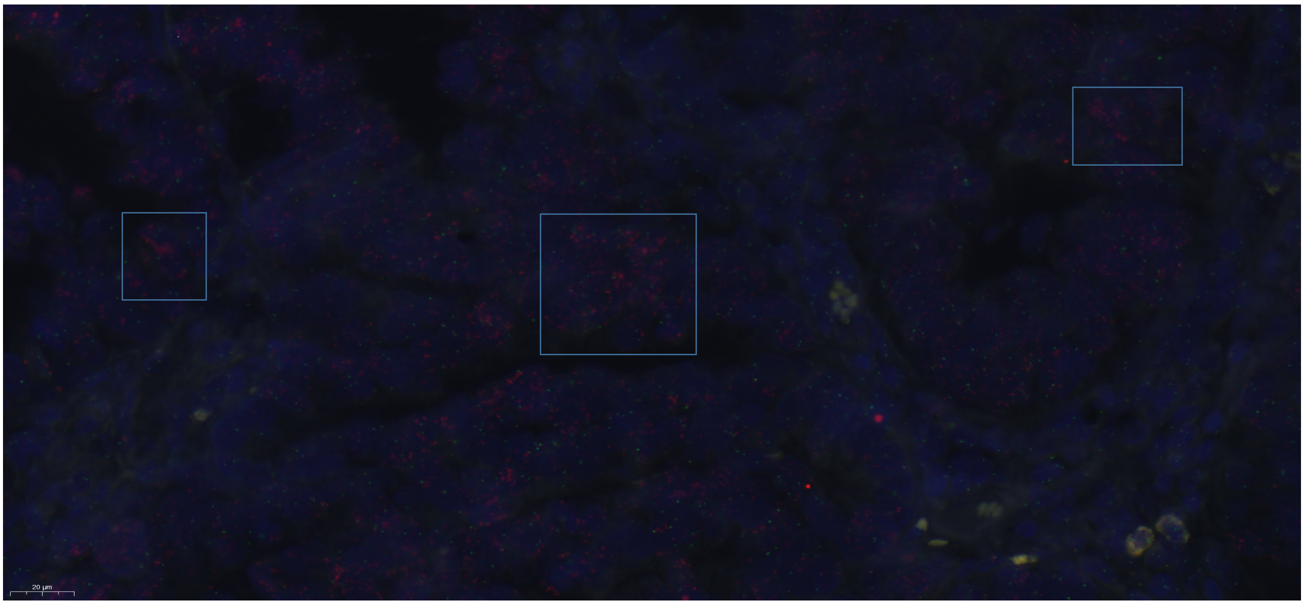

Supplementary Figure 23. CRC978, Male, *MYC* (63X).

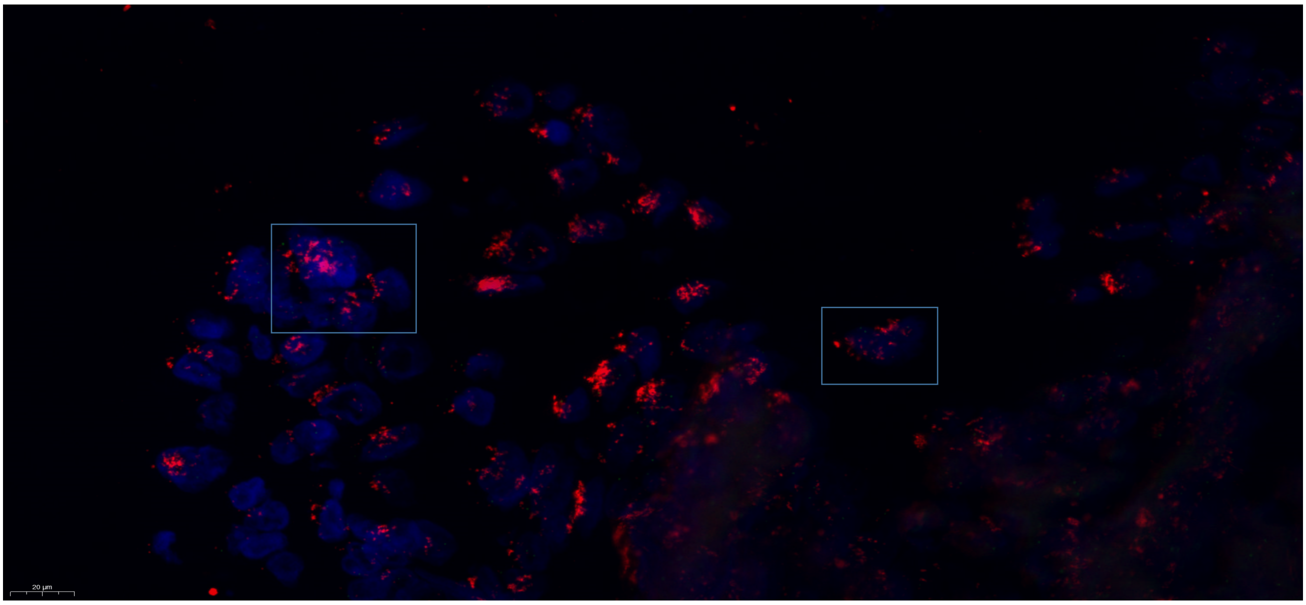

Supplementary Figure 24. CRC875, Female, *MYC* (63X).

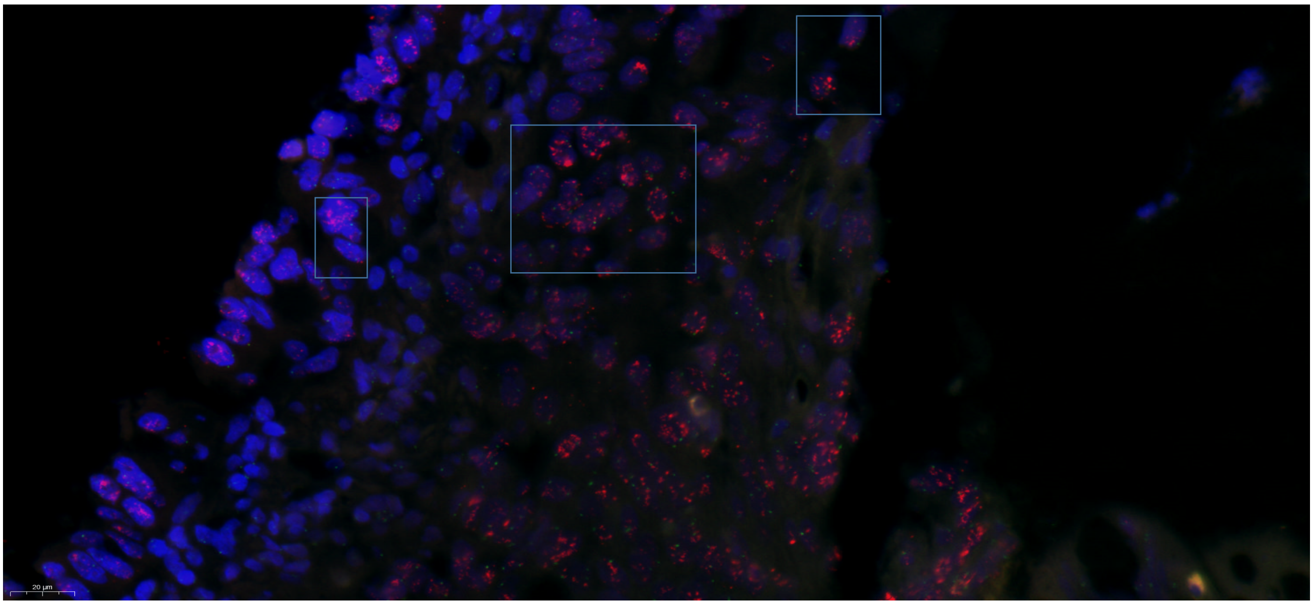

Supplementary Figure 25. CRC938, Female, *MYC* (63X).

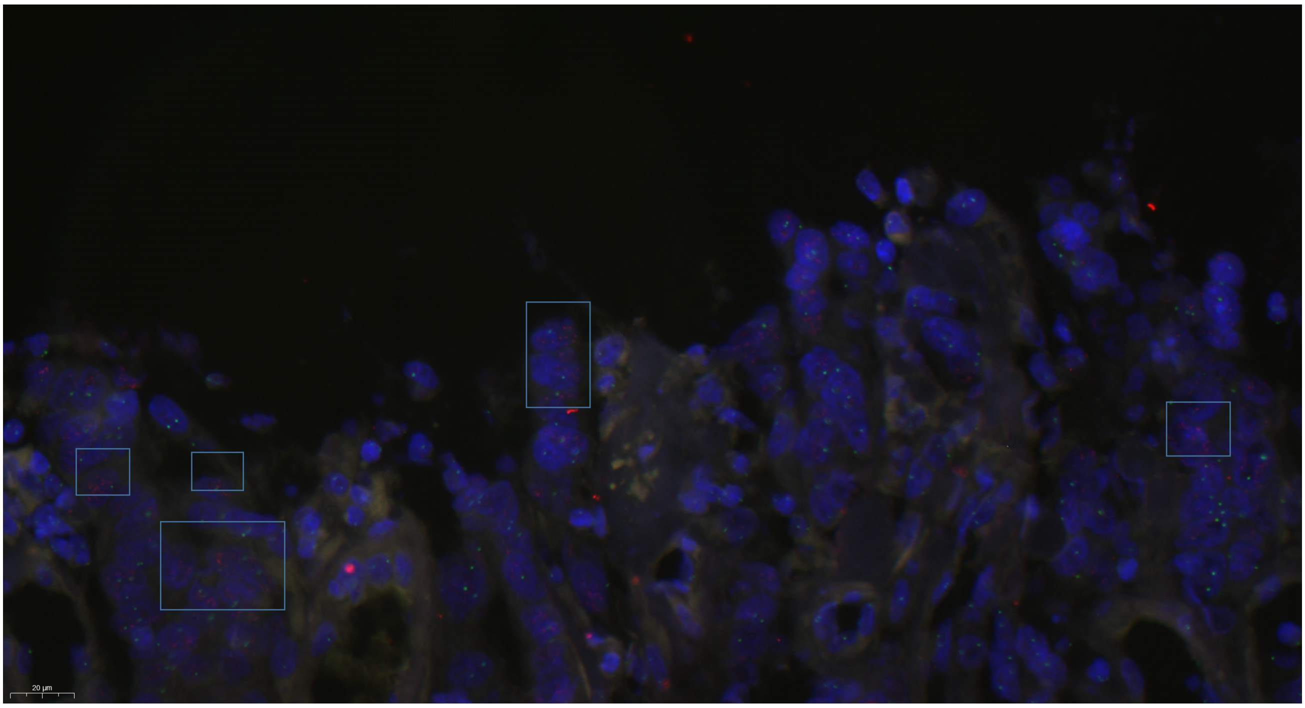

Supplementary Figure 26. CRC938, Female, *ERBB2* (63X).

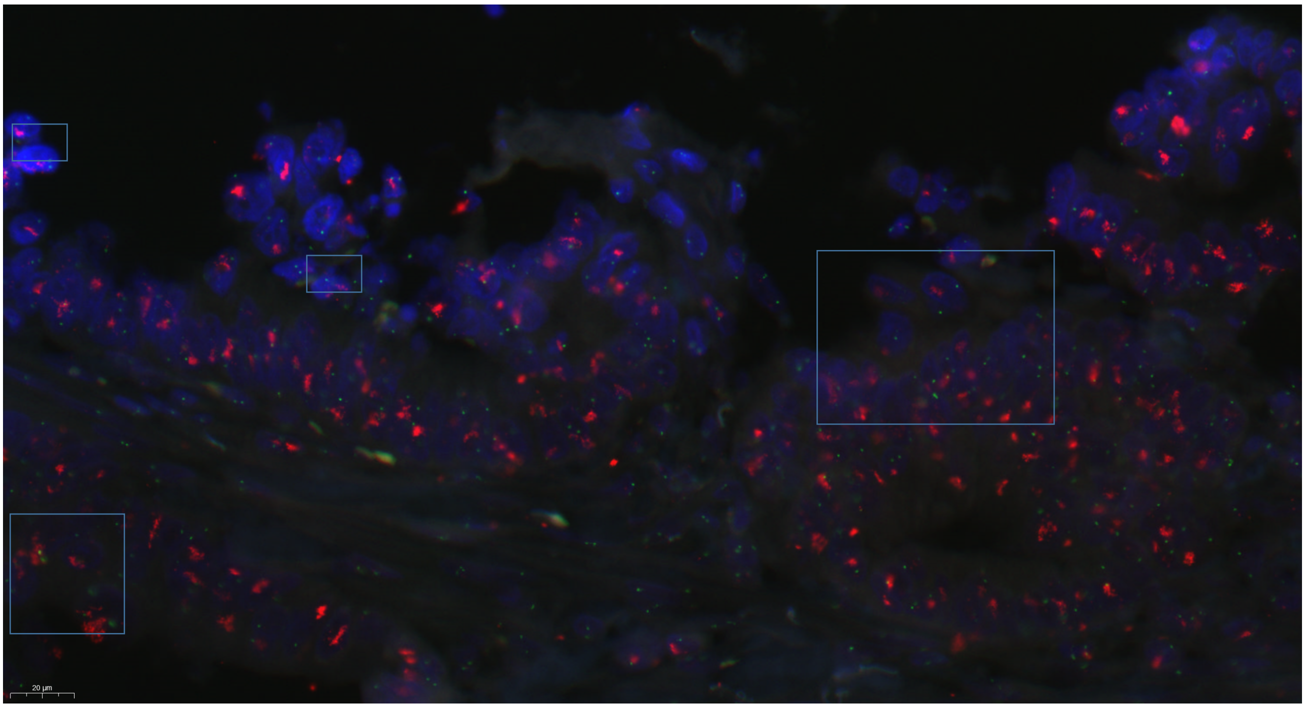

Supplementary Figure 27. CRC983, Male, *ERBB2* (63X).

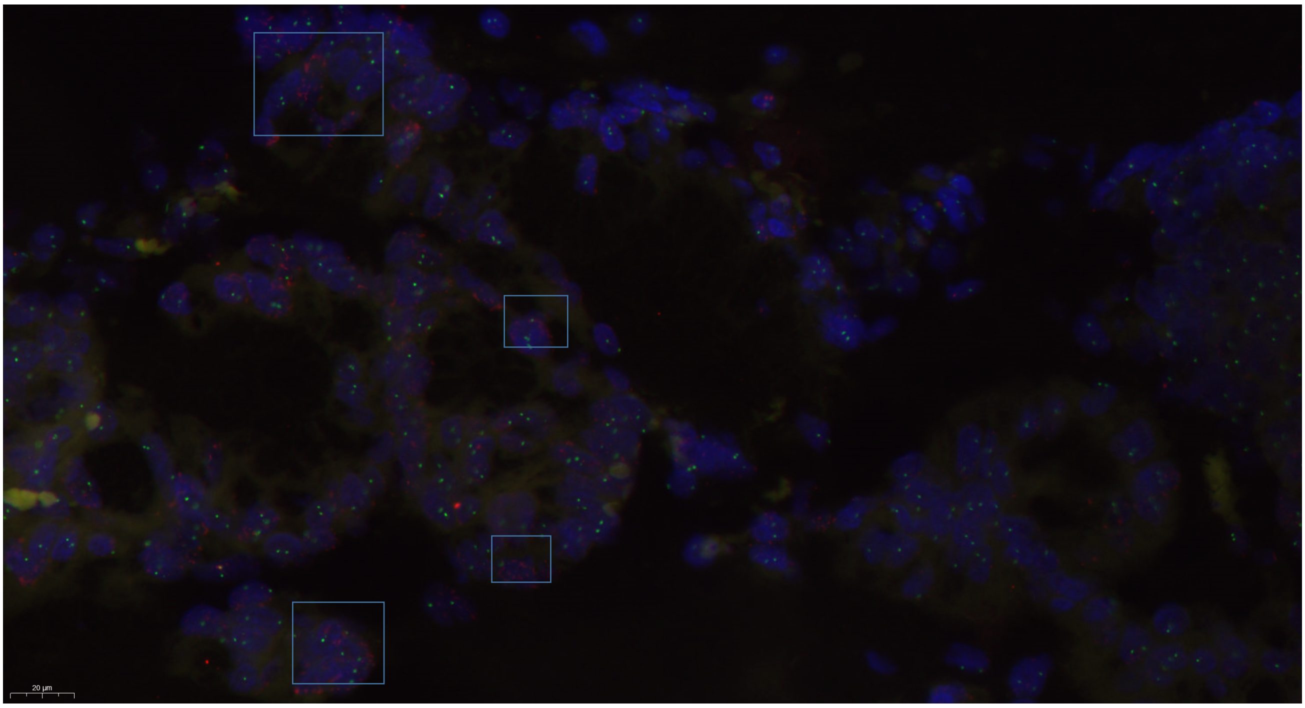

Supplementary Figure 28. CRC634, Female, *ERBB2* (63X).

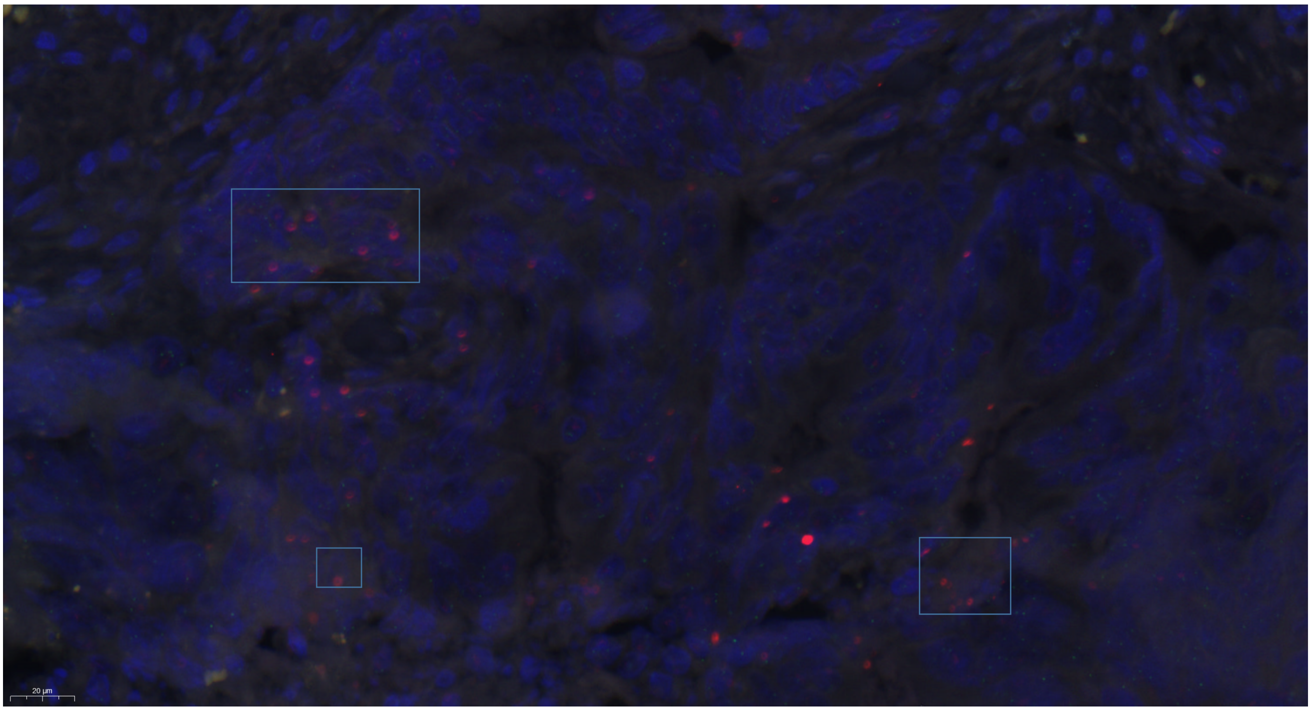

Supplementary Figure 29. CRC211, Male, *ERBB2* (63X).

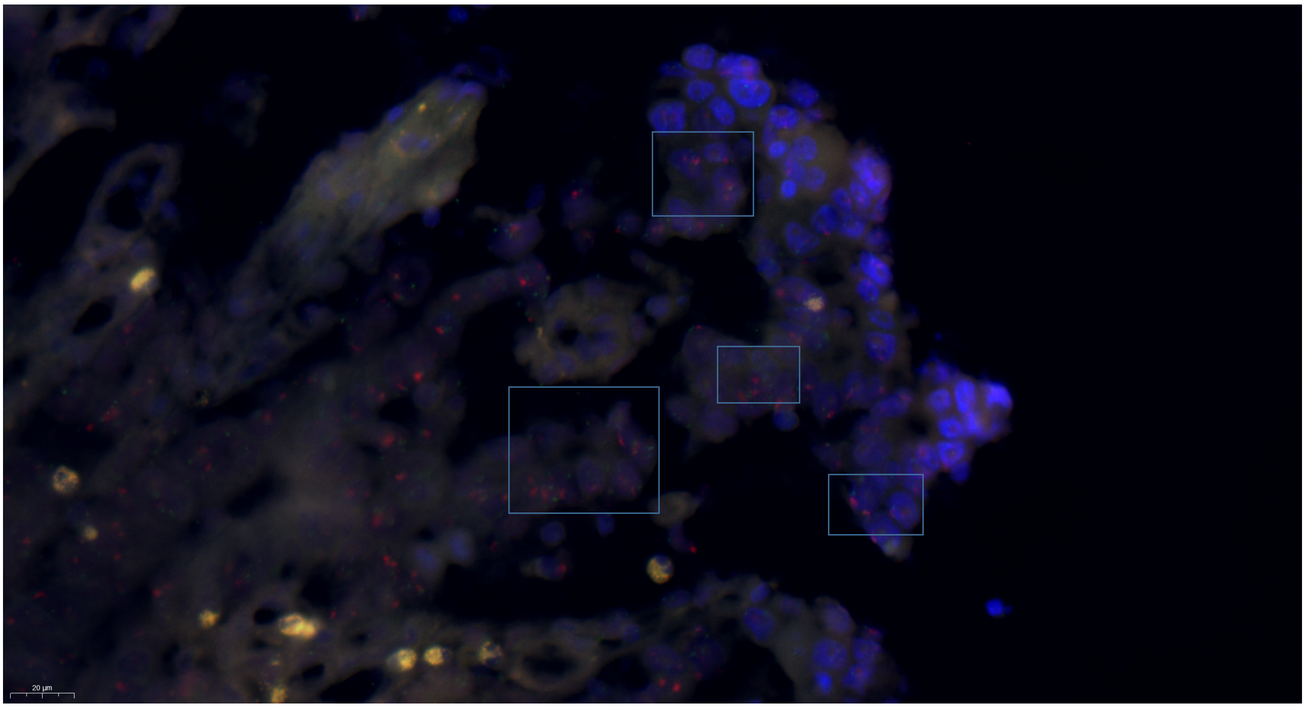

Supplementary Figure 30. CRC259, Male, *ERBB2* (63X).

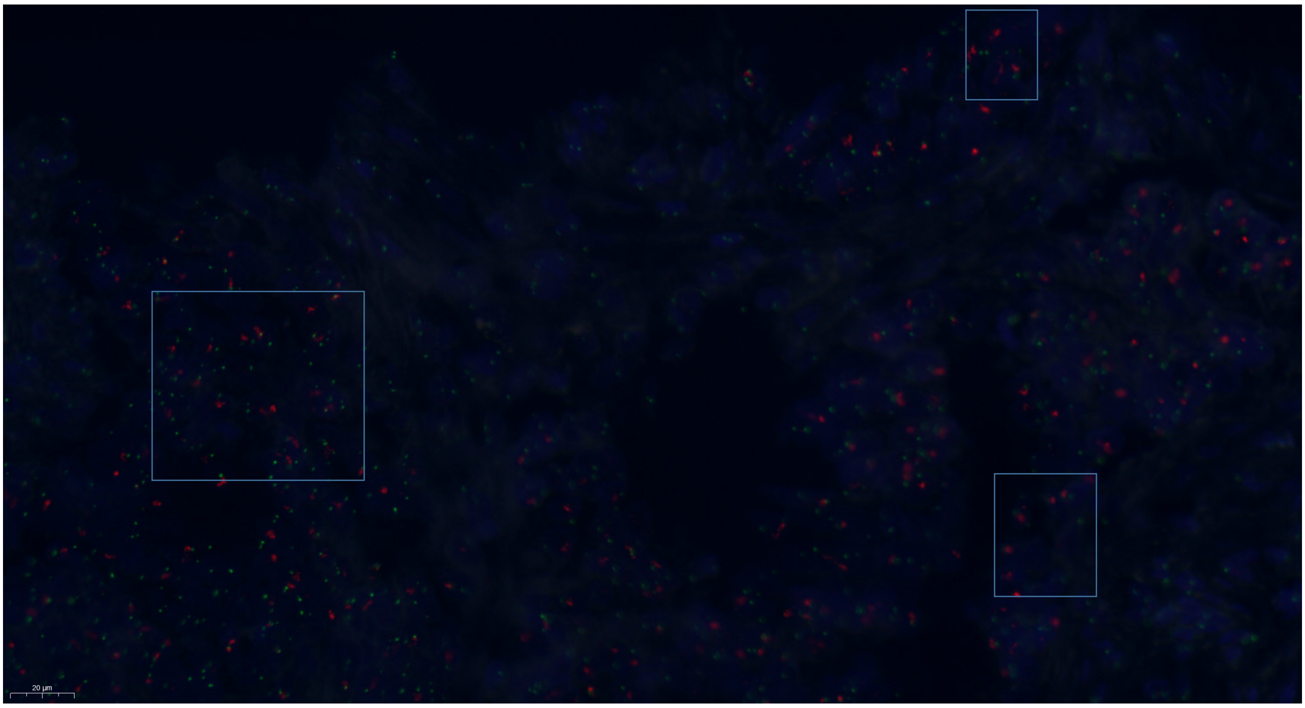

Supplementary Figure 31. CRC350, Female, *ERBB2* (63X).

AmpliconArchitect results

In FISH images, *ERBB2* amplification commonly manifests as a pattern of locally clustered gene amplification signals. Considering the reduced confidence in the FISH results, which may be more susceptible to false positives in detecting ecDNA presence, we collected frozen tissue sections from the corresponding patients for whole-genome sequencing (averaging a sequencing depth of 10x) and AmpliconArchitect analysis. The summarized results, along with the classification radar plots, are provided below. All result files of AA analysis are available at https://zenodo.org/records/10212116/files/aa_ffpe.zip.

Supplementary Table 1. AmpliconArchitect result summary table.

Sample	amplicon_decomposition_class	Feature ID	Classification	Oncogenes	Feature median copy number
CRC211	Cyclic	CRC211_amplicon1_BFB_1	BFB	['CDC6', 'CSF3', 'ERBB2', 'RARA', 'THRA']	83.12
CRC259	Cyclic	CRC259_amplicon1_ecDNA_1	ecDNA	['ERBB2']	52.45
CRC259	Cyclic	CRC259_amplicon2_ecDNA_1	ecDNA	[]	21.00
CRC259	Cyclic	CRC259_amplicon2_BFB_1	BFB	[]	5.25
CRC350	Cyclic	CRC350_amplicon1_ecDNA_1	ecDNA	['CDK12', 'ERBB2']	16.81
CRC634	Complex non-cyclic	CRC634_amplicon1_Complex non-cyclic_1	Complex non-cyclic	['ERBB2']	9.39
CRC938	Cyclic	CRC938_amplicon1_ecDNA_1	ecDNA	['ERBB2', 'MYC', 'PVT1']	14.18
CRC983	Cyclic	CRC983_amplicon1_BFB_1	BFB	['CDK12', 'ERBB2']	17.33

NOTE: full table could be found at https://zenodo.org/records/10212116/files/AA_summary_table_of_6_erbb2_ffpe_samples.xlsx.

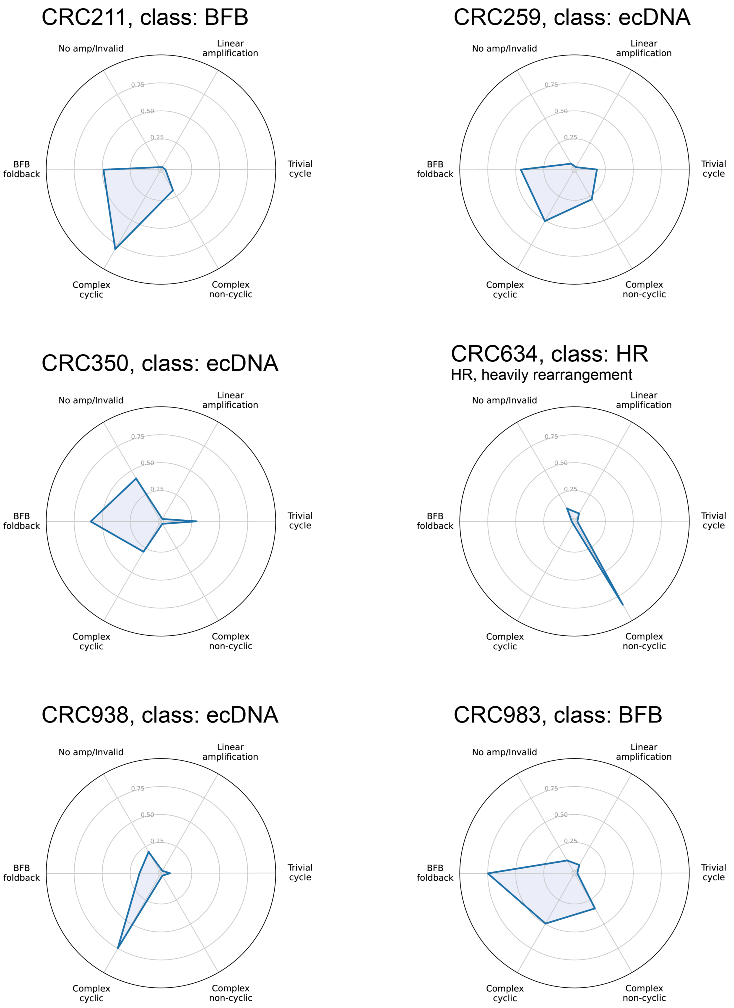

Supplementary Figure 32. Radar-style plots of amplicon classification strengths.

The plots were generated by AmpliconClassifier (https://github.com/AmpliconSuite/AmpliconClassifier).
