# Supplementary material for: Machine learning-based extrachromosomal DNA identification in large-scale cohorts reveals its clinical implications in cancer
Source: Nat Commun. 2024 Feb 19;15:1515. doi: 10.1038/s41467-024-45479-6 (PMC10876971; doi:10.1038/s41467-024-45479-6)
Supplement: Supplementary file 3 — Description of Additional Supplementary Files [file 41467_2024_45479_MOESM3_ESM.pdf]

Description of Additional Supplementary Files

File Name: Supplementary Data 1
Description: Classification comparison between AmpliconArchitect and GCAP in WGS of 40 cancer cell lines.

File Name: Supplementary Data 2
Description: High-quality circular DNA regions identified by Circle-Map from Circle-Seq data of 11 cancer cell lines.

File Name: Supplementary Data 3
Description: Association analysis results between ecDNA and oncogenes in the TCGA database.

File Name: Supplementary Data 4
Description: Mutational signature enrichment analysis results for six genomic subtypes in the SYSUCC CRC cohort.

File Name: Supplementary Data 5
Description: Feature summary table of survival risk levels, mutational processes, and etiologies for six genomic subtypes of colorectal cancer.
