# Supplementary material for: Machine learning-based extrachromosomal DNA identification in large-scale cohorts reveals its clinical implications in cancer
Source: Nat Commun. 2024 Feb 19;15:1515. doi: 10.1038/s41467-024-45479-6 (PMC10876971; doi:10.1038/s41467-024-45479-6)
Supplement: Supplementary file 9 — Reporting Summary [file 41467_2024_45479_MOESM9_ESM.pdf]

nature portfolio

Corresponding author(s): Qi Zhao

Last updated by author(s): Dec 18, 2023

Reporting Summary

Nature Portfolio wishes to improve the reproducibility of the work that we publish. This form provides structure for consistency and transparency in reporting. For further information on Nature Portfolio policies, see our Editorial Policies and the Editorial Policy Checklist.

Statistics

For all statistical analyses, confirm that the following items are present in the figure legend, table legend, main text, or Methods section.

n/a	Confirmed	
☐	☒	The exact sample size (*n*) for each experimental group/condition, given as a discrete number and unit of measurement
☐	☒	A statement on whether measurements were taken from distinct samples or whether the same sample was measured repeatedly
☐	☒	The statistical test(s) used AND whether they are one- or two-sided<br>*Only common tests should be described solely by name; describe more complex techniques in the Methods section.*
☐	☒	A description of all covariates tested
☐	☒	A description of any assumptions or corrections, such as tests of normality and adjustment for multiple comparisons
☐	☒	A full description of the statistical parameters including central tendency (e.g. means) or other basic estimates (e.g. regression coefficient) AND variation (e.g. standard deviation) or associated estimates of uncertainty (e.g. confidence intervals)
☐	☒	For null hypothesis testing, the test statistic (e.g. *F*, *t*, *r*) with confidence intervals, effect sizes, degrees of freedom and *P* value noted<br>*Give P values as exact values whenever suitable.*
☒	☐	For Bayesian analysis, information on the choice of priors and Markov chain Monte Carlo settings
☐	☒	For hierarchical and complex designs, identification of the appropriate level for tests and full reporting of outcomes
☐	☒	Estimates of effect sizes (e.g. Cohen's *d*, Pearson's *r*), indicating how they were calculated

Our web collection on statistics for biologists contains articles on many of the points above.

Software and code

Policy information about availability of computer code

Data collection	gdc-client v1.6.1, Oncogene database (http://ongene.bioinfo-minzhao.org/), GitHub (https://github.com/VanLoo-lab/ascat/tree/master/ReleasedData/TCGA_SNP6_hg19), PCAWG Xena hub (https://pcawg.xenahubs.net), Pan-Cancer Atlas hub (https://pancanatlas.xenahubs.net), COSMIC (https://cancer.sanger.ac.uk/signatures/sbs/), GSA-Human (https://ngdc.cncb.ac.cn/gsa-human/browse/HRA000873), NCBI SRA database.
Data analysis	Circle-Map v1.1.4 (https://github.com/iprada/Circle-Map), ASCAT v3.0 (https://github.com/VanLoo-lab/ascat), AmpliconArchitect (https://nf-co.re/circdna, v1.0.2), CNVkit v0.9.9 (https://github.com/etal/cnvkit), Sigminer v2.2.0 (https://github.com/ShixiangWang/sigminer), GCAP v1.0.0 (https://github.com/ShixiangWang/gcap), GCAPutils v0.0.0.9000 (https://github.com/ShixiangWang/gcaputils), R v4.0.2 (https://cran.r-project.org/). Analysis code is available at https://github.com/ShixiangWang/gcap-wes.

For manuscripts utilizing custom algorithms or software that are central to the research but not yet described in published literature, software must be made available to editors and reviewers. We strongly encourage code deposition in a community repository (e.g. GitHub). See the Nature Portfolio guidelines for submitting code & software for further information.

Data

Policy information about availability of data

All manuscripts must include a data availability statement. This statement should provide the following information, where applicable:

- Accession codes, unique identifiers, or web links for publicly available datasets
- A description of any restrictions on data availability
- For clinical datasets or third party data, please ensure that the statement adheres to our policy

Human oncogene list was obtained from the Oncogene database (http://ongene.bioinfo-minzhao.org/). TCGA tumor-normal paired WES data from 386 pan-cancer samples (sample list is available in Source Data file) for gene prediction modeling were downloaded from GDC data portal with gdc-client v1.6.1 (dbGaP accession number phs000178.v9.p8 [https://www.ncbi.nlm.nih.gov/projects/gap/cgi-bin/study.cgi?study_id=phs000178.v9.p8]). TCGA allele specific copy number profiles can be found at https://github.com/VanLoo-lab/ascat/tree/master/ReleasedData/TCGA_SNP6_hg19. PCAWG allele specific copy number profiles and survival data can be found at PCAWG Xena hub (https://pcawg.xenahubs.net). Other types of data including gene expression, mutation, survival data for TCGA are available at Pan-Cancer Atlas hub (https://pancanatlas.xenahubs.net). The raw sequence data of Changkang Project have been deposited in the Genome Sequence Archive in National Genomics Data Center, China National Center for Bioinformation / Beijing Institute of Genomics, Chinese Academy of Sciences, under accession number HRA000873 [https://ngdc.cncb.ac.cn/gsa-human/browse/HRA000873]. The processed clinical annotations and structured genomic dataset for Changkang Project are available at Zhao et al. and https://changkang.hapyun.com/. SRA accessions of WGS data for cancer cell lines were collected in Supplementary Data 1. WES and Circle-Seq data for cancer cell lines generated by this study were deposited in SRA BioProject under accession number PRJNA894840 [https://www.ncbi.nlm.nih.gov/bioproject/PRJNA894840]. The raw sequencing data related to PDX/clinical samples are protected and are not available due to data privacy laws. The processed data for ecDNA cargo gene modeling, GCAP and AmpliconArchitect results for cancer cell lines, TCGA, PCAWG and Changkang Project, PDX/clinical samples, etc. were deposited in Zenodo (https://zenodo.org/doi/10.5281/zenodo.7272630) with open access. The remaining data are available in the Supplementary Information or Source Data file.

Research involving human participants, their data, or biological material

Policy information about studies with human participants or human data. See also policy information about sex, gender (identity/presentation), and sexual orientation and race, ethnicity and racism.

Reporting on sex and gender	Samples from two male patients with gastric cancer were collected and investigated in this study. For public cancer databases (i.e., TCGA and PCAWG) and clinical cancer cohorts, the available sex or other information have been published in previous corresponding studies.
Reporting on race, ethnicity, or other socially relevant groupings	The gastric cancers in this study were collected from Asian race (Chinese) people. We did not collected other socially relevant groupings information. The study did not involve relevant analyses as they are out of our study foucs.
Population characteristics	Multiple cohorts were included in the study: TCGA (N = 9699), PCAWG (N = 2778), SYSUCC CRC (N = 1015), SYSUCC NPC (N = 170), SYSUCC AGC (N = 55), SKKU AGC (N = 55) and JUPITER-06 (N = 486). Focal amplification profiles of these cohorts were analyzed. Other characteristics (e.g., age, metastasis, treatment response, survival) were analyzed and reported based on study design for illustrating the findings.
Recruitment	The research is a genomically descriptive research based on published datasets, so no recruitment in the research.
Ethics oversight	The Institutional Review Board of Sun Yat-Sen University Cancer Center

Note that full information on the approval of the study protocol must also be provided in the manuscript.

Field-specific reporting

Please select the one below that is the best fit for your research. If you are not sure, read the appropriate sections before making your selection.

☒ Life sciences ☐ Behavioural & social sciences ☐ Ecological, evolutionary & environmental sciences

For a reference copy of the document with all sections, see nature.com/documents/nr-reporting-summary-flat.pdf

Life sciences study design

All studies must disclose on these points even when the disclosure is negative.

Sample size	The research is a descriptive research, so no statistical methods were used to determine the sample size. As ~20% cancer patients across pan-cancer types have ecDNA amplification, we validated our developed GCAP with the most well-known pan-cancer studies TCGA (N = 9699), PCAWG (N = 2778), then used multiple cohorts with a range of sample size, including SYSUCC CRC (N = 1015), SYSUCC NPC (N = 170), SYSUCC AGC (N = 55), SKKU AGC (N = 55) and JUPITER-06 (N = 486), for discovery.
Data exclusions	No data were excluded from the analyses.
Replication	The processed data are available in Zenodo (https://doi.org/10.5281/zenodo.8373312) or this manuscript, and the analysis code is deposited in GitHub (https://github.com/ShixiangWang/gcap-wes).

Randomization	The research is a genomically descriptive research based on published datasets, so no allocation and randomization was used in the research. In general, we used our developed tool GCAP to assign three different classes (nofocal, noncircular and circular) to clinical cancers for research purpose. In some cases, we also combined our GCAP determined classes with predefined patient groups in previous studies to generated refined subtypes. We analyzed the survival data with Cox regression model for considering the reported/common clinical/ molecular covariates.
Blinding	The research is a genomically descriptive research based on published datasets, so no allocation and blinding was used in the research. We were not blinded to the sample group allocation for collecting modeling data as machine learning requires known patient group labels. For analyzing data in multiple cohorts, we applied our developed tool GCAP to automatically determine the patient groups for comparison or other research purpose.

Reporting for specific materials, systems and methods

We require information from authors about some types of materials, experimental systems and methods used in many studies. Here, indicate whether each material, system or method listed is relevant to your study. If you are not sure if a list item applies to your research, read the appropriate section before selecting a response.

Materials & experimental systems

n/a	Involved in the study
☒	☐ Antibodies
☐	☒ Eukaryotic cell lines
☒	☐ Palaeontology and archaeology
☐	☒ Animals and other organisms
☒	☐ Clinical data
☒	☐ Dual use research of concern
☒	☐ Plants

Methods

n/a	Involved in the study
☒	☐ ChIP-seq
☒	☐ Flow cytometry
☒	☐ MRI-based neuroimaging

Eukaryotic cell lines

Policy information about cell lines and Sex and Gender in Research

Cell line source(s)	SNU16, PC3, KYSE410, OE19, SNU216, HGC27, KATOIII, MKN7, MKN45, MKN74, NCIN87 cell lines were purchased from ATCC.
Authentication	The cell lines were identified by short tandem repeat (STR) markers—tiny repeating segments of DNA found between genes —at specific loci to establish a DNA fingerprint for every human cell line.
Mycoplasma contamination	Tested negative for mycoplasma.
Commonly misidentified lines (See ICLAC register)	No.

Animals and other research organisms

Policy information about studies involving animals; ARRIVE guidelines recommended for reporting animal research, and Sex and Gender in Research

Laboratory animals	All mice used in this study were NOD/SCID/IL2rγnull (NSG) female mice (6 weeks). Mice were housed under temperature-controlled, pathogen-free conditions (approximately 20°C, 40% humidity) with a 12-h light/dark cycle. Small pieces of tissue (1–3 mm3) were directly implanted into bilateral subcutaneous pockets of NSG mice. In the initial passage of PDX, the tumor reached a volume of about 500 mm3 and then was transplanted into other mice (P2). PDX of different generations was reserved and placed in liquid nitrogen along with tissue preservation solution
Wild animals	The study did not involve wild animals.
Reporting on sex	Female mice were used. Since our experimental design did not involve sex-related factors and it was previously shown that the gender of SCID mice did not affect PDX model development, the following experiments were performed using only female mice.
Field-collected samples	The study did not involve field-collected samples.
Ethics oversight	The Institutional Ethics Committee for Clinical Research and Animal Trials of the SYSUCC approved all animal studies

Note that full information on the approval of the study protocol must also be provided in the manuscript.
